# Role of SARS-CoV-2 Spike-Protein-Induced Activation of Microglia and Mast Cells in the Pathogenesis of Neuro-COVID

**DOI:** 10.3390/cells12050688

**Published:** 2023-02-22

**Authors:** Theoharis C. Theoharides, Duraisamy Kempuraj

**Affiliations:** 1 Institute for Neuro-Immune Medicine, Dr. Kiran C. Patel College of Osteopathic Medicine, Nova Southeastern University, Fort Lauderdale, FL 33328, USA; 2 Laboratory of Molecular Immunopharmacology and Drug Discovery, Department of Immunology, Tufts University School of Medicine, Boston, MA 02111, USA

**Keywords:** ACE2, brain, coronavirus, cytokines, inflammation, microglia, spike protein, toll-like receptors

## Abstract

Severe acute respiratory syndrome coronavirus 2 (SARS-CoV-2) causes coronavirus disease 2019 (COVID-19). About 45% of COVID-19 patients experience several symptoms a few months after the initial infection and develop post-acute sequelae of SARS-CoV-2 (PASC), referred to as “Long-COVID,” characterized by persistent physical and mental fatigue. However, the exact pathogenetic mechanisms affecting the brain are still not well-understood. There is increasing evidence of neurovascular inflammation in the brain. However, the precise role of the neuroinflammatory response that contributes to the disease severity of COVID-19 and long COVID pathogenesis is not clearly understood. Here, we review the reports that the SARS-CoV-2 spike protein can cause blood–brain barrier (BBB) dysfunction and damage neurons either directly, or via activation of brain mast cells and microglia and the release of various neuroinflammatory molecules. Moreover, we provide recent evidence that the novel flavanol eriodictyol is particularly suited for development as an effective treatment alone or together with oleuropein and sulforaphane (ViralProtek^®^), all of which have potent anti-viral and anti-inflammatory actions.

## 1. Introduction

Severe acute respiratory syndrome coronavirus 2 (SARS-CoV-2) causes coronavirus disease 2019 (COVID-19), leading to complex immune responses [1,2] that involve the release of several inflammatory cytokines/chemokines [1,3,4,5,6,7,8], especially interleukin-1beta (IL-1β) [9] and IL-6, often referred to as “cytokine exacerbated production” [3,10,11]. Almost 50 percent of patients infected with SARS-CoV-2 experience post-acute sequelae of SARS-CoV-2 (PASC) [12,13,14] shortly after the initial infection [15], known as “Long-COVID syndrome” [16,17,18]. A recent report indicated that about 45% of COVID-19 survivors showed persistent symptoms at about 4 months in post COVID-19 population, with fatigue being most frequently experienced in hospitalized and non-hospitalized cohorts [14]. Long COVID is characterized by persistent fatigue that is not dependent on the initial severity of the disease [19] and presents with persistent symptomatology many months post-acute infection [20]. At least 20–45 percent of COVID-19 survivors experience various neuropsychiatric [14,21,22,23,24,25,26,27,28,29,30,31,32,33], neurological [34,35,36,37,38,39,40,41,42] and neurodegenerative [37,43] issues, sleep disturbances [44], and cognitive deficits [28,29,30,31,32,33,45,46,47,48], especially brain fog [16,17,49,50,51,52,53,54,55]. The length of long COVID may depend on the persistence of some viral antigens [56] and the magnitude of continued inflammatory reactions to SARS-CoV-2 [57]. Long COVID has been considered as the “Next national health disaster” for the US [58] that could have a “$3.7 trillion economic impact rivaling the Great Depression” [59].

A systematic review of the literature using the MEDLINE data base (1 January 1990–1 January 2023) was conducted to identify peer-reviewed publications relevant to the diagnosis, pathogenesis and treatment of neuro-COVID using the search terms angiotensin-converting enzyme 2 (ACE2), blood-brain barrier (BBB), brain, chemokines, corona virus, COVID-19, cytokines, endothelial cells, fatigue, fog, inflammation, Long-COVID, mast cell, microglia, neuroinflammation, toll-like receptors, and vasculature. Emphasis was placed on publications reporting original data, especially in humans, even though reviews and papers using animal models were also included.

We advance the premise that brain perivascular inflammation is a critical pathogenetic factor in long COVID mostly due to SARS-CoV-2 activating brain mast cells and microglia resulting in the release of inflammatory, neurotoxic, and vasoactive mediators [60,61].

## 2. Long COVID Pathogenesis Is Unknown

The precise mechanism of long COVID pathogenesis has yet to be fully elucidated [62]. It is well-understood that SARS-CoV-2 enters cells through the coronavirus spike (S) protein binding to its cell surface receptor, ACE2 [63]. How SARS-CoV-2 enters the brain is not yet clearly known, but increased levels of cytokines were reported in the cerebrospinal fluid (CSF) of patients with COVID-19 [64,65]. The virus may enter the brain from the nose through the nasal neural mucosa [66] following the olfactory nerve tract [67] or via the gustatory–olfactory trigeminal pathway and cause BBB dysfunction. 

Autopsy from a deceased infant with COVID-19 showed severe neuronal loss in the capillaries of the choroid plexus [68]. Another autopsy study detected choroid plexus morphological changes in the microglia [69,70]_,_ as well as neuronal necrosis and glial cell hyperplasia in the brain of a deceased patient with COVID-19 [71]. While the exact pathogenetic mechanisms [50] remain unclear, evidence points to the involvement of neuroinflammation [72,73,74], and neurovascular inflammation that can damage brain blood vessels [75,76] and brain cells [72,77,78].

### Role of SARS-CoV-2 S Protein in the Nervous System Damage

The neurological issues of long COVID [79] may be attributed to the entry of SARS-CoV-2 into the brain [80], but the routes of viral entry are not yet clear [81]. The S protein is involved in the fusion of the viral membrane with the surface membrane of the host. The S trimer structure has three receptor-binding domains (RBD), while the post-fusion structure expresses N-linked glycans that may protect the immune system [82]. Recent evidence and our studies indicate that the spike protein can also directly activate microglia [83,84,85], leading to proinflammatory effects [86] and microglia–synapse elimination [87]. SARS-CoV-2 can also lead to brain vascular damage and endothelial dysfunction [88,89,90,91], BBB disruption [92,93,94,95,96,97,98] and reduced blood flow to the brain [99]. A recent study reported that neuroinflammation could disrupt the “blood-central nervous system (CNS) barrier” in a mouse model of multiple sclerosis (MS) that involved the interaction of inflammatory, endothelial, and mesenchymal pathways [100]. Perivascular inflammation with lymphocytic and microglial infiltration was noted in the brains of 52 deceased patients with COVID-19 [101]. A cross-sectional study identified BBB disruption along with increased microglial activation markers and increased B-cell responses against self and non-self-antigens [102]. Another study with 24 neuro-COVID patients also reported increased intrathecal immunoglobulin, neopterin, and neurofilament light chain (NfL) levels [103]. Apolipoprotein E4 (ApoE4) has been associated with COVID-19 [104,105,106] and with severe COVID-19 [100,101]. In fact, ApoE4 could possibly predict COVID-19 pathogenesis [107]. In particular, ApoErs429358 polymorphism was associated with an increased risk of COVID-19 infection [108]. Elevated ApoE4 levels were reported to also reflect BBB disruption and predict cognitive decline [109].

SARS-CoV-2 has been reported to activate toll-like receptors (TLRs) [110,111] leading to the release of immune molecules that could contribute to neurologic symptoms [112]. TLRs are important in recognizing viral particles and orchestrate innate immune responses. Viral activation of TLRs causes the release of inflammatory cytokines from immune cells [113]. Microglia have many receptors, including TLRs [114], and they are activated by damage-associated molecular patterns (DAMPs) and pathogen-associated molecular patterns (PAMPs) [114]. TLRs were recently shown to mediate COVID-19 pathogenesis [115]. One paper reported that SARS-CoV-2 envelope protein could produce inflammatory cytokines from mouse bone-marrow-derived macrophages via TLR2 activation, independent of viral entry [116]. Another study demonstrated that SARS-CoV-2 S protein could stimulate BV-2 microglia leading to the release of interleukin-1 beta (IL-1β), IL-6, and tumor necrosis factor-alpha (TNF-α) with increased expression of TLR4 [84]. Another paper reported that infection of HMC3 microglia also led to the release of IL-1β, IL-6, and TNF-α [83]. Moreover, activation of TLR4 increased the expression of ACE2 [117], further enhancing viral infectivity in an autocrine loop fashion. In fact, TLR4 has been considered as a therapeutic target for neurological complications associated with SARS-CoV-2 infection [118,119]. Increased levels of pro-inflammatory cytokines, especially IL-6, have been detected in the CSF of COVID-19 patients [65] and have been implicated in neurologic diseases associated with COVID-19 [64]. Our recent findings show that SARS-CoV-2 can stimulate human microglia to secrete distinct pro-inflammatory mediators via activation of different receptors: recombinant whole-length S protein results in secretion of IL-1β and chemokine (C-X-C motif) ligand 8 (CXCL8) not via activation of ACE2, but rather activation of TLR-4, while the recombinant receptor binding domain (RBD) of the S protein stimulates the release of IL-18, TNF-α, and S100B via ACE2 [120].

## 3. Microglia-Induced Neuroinflammation and Mental Health

Microglia are specialized macrophage-like immune cells of the CNS and constitute about 7 percent of non-neuronal cells in the brain [121]. It has been reported that one microglial cell serves 1 to 100 neuronal cells in various brain areas with different neuronal densities [121]. Microglia are important for CNS homeostasis both in health and disease states [122], especially neurodegenerative [123,124,125,126,127,128,129] and neuroinflammatory [122,128,130,131] diseases, including COVID-19 [83,132]. During neuroinflammatory response and brain homeostasis maintenance, microglia can change their numbers, morphological characteristics, molecular pattern, and functions [132]. Activated microglia release pro-inflammatory cytokines, free radicals, and fatty acid metabolites. Cytokines and chemokines released from activated microglia induce activation of astrocytes with additional release of proinflammatory mediators that further exacerbates neuroinflammatory response. Dysregulated microglia and T-cell interactions and microglial nodules in the perivascular compartment of the brain were associated with systemic inflammatory conditions in COVID-19 [133]. Microglial activation is significantly higher in the brain stem than in non-COVID cases. Further, COVID-19 cases without dementia show more microglial activation in the brain stem [134,135]. The neuroinflammatory response is indicated by the presence of microglial reactivity indicators such as CD68-positive ameboid microglia, ionized calcium binding adaptor molecule 1 (IBA1), and human leukocyte antigen-DR (HLA-DR) in COVID-19 [132,134]. COVID-19 shows more T lymphocytes and microthromboses in the lung associated with more microglial activation in the brain stem [135]. In other words, the long-term consequences of COVID-19 could be due to persistent inflammation rather than persistent viral replication [135]. SARS-CoV-2 induces neuropsychiatric and neurological disorders such as cognitive decline, depression, dizziness, delirium, and sleep disorders that lead to neuronal damage, neurodegenerative disorders, and dementia [136]. Thus, SARS-CoV-2 can cause BBB disruption and worsen neurodegenerative diseases such as Alzheimer’s disease (AD) and Parkinson’s disease, especially in aged people [136,137,138].

SARS-CoV-2 infection can cause dysregulation of the hypothalamic–pituitary–adrenal (HPA) axis [139], which may be the cause of the emotional changes observed during and after viral infection [140]. Several reports have shown the impact of the pandemic on acute and chronic mental health. Further, these studies also focused on the psycho-social factors and stress resilience of mental health and disease pathologies [141,142]. TLR4 contributes to the immune response and pathogenesis of COVID-19, and thus, TLR4 could be a therapeutic target in COVID-19 [113,143,144]. SARS CoV-2 activates TLR4 and 8 and induces cytokine release from microglia and monocytes [145]. Microglia express receptors for neurotensin (NT) [146] and corticotropin-releasing hormone (CRH), secreted under stress [147], which are especially associated with COVID-19 [148]. Microglia are typically characterized as resting (M0), pro-inflammatory (M1), and anti-inflammatory and neuroprotective (M2) phenotypes with different cytokine expressions associated with neuroinflammatory response. We reported that cultured human microglia can be activated by neuropeptides such as NT to release IL-1β and CXCL8 [149] that induces proinflammatory response. Microglial-derived proinflammatory cytokines and chemokines induce astrogliosis, amyloid deposition, and subsequently, further worsening neuroinflammation [122]. Psychological stress can increase microglial reactivity to other challenges [150] and lead to cognitive decline [151] and neuroinflammatory response.

Microglia are increasingly involved in the pathogenesis of psychiatric disorders [132,152,153]. In fact, microglia-induced neuroinflammation was considered a risk factor for the pathogenesis of major depressive disorder [154,155]. Moreover, SARS-CoV-2 neurotropism may increase the severity of neuropsychiatric issues [156]. A recent report indicated that the SARS-CoV-2 protein elicited a robust nuclear factor kappa B (NF-κB)/nucleotide-binding domain (NOD)-like receptor protein 3 (NLRP3) inflammasome-mediated pro-inflammatory response and increased Iba1 expression in a BV-2 mouse microglial cell line [84]. In addition, post-mortem reports of COVID-19 patients showed significant microglial activation and neuroinflammation associated with brain pathology [157,158,159,160]. Increasing reports indicate that elevated inflammatory cytokines and neuroinflammatory responses [72,128,161] can damage brain blood vessels [75,162] and other brain cells [72,77,78], possibly through abnormally excessive activation of microglia [60,61]. As such, long COVID could be referred to as “brain autoimmunity” [163].

## 4. Microglia Communicate with Mast Cells

**Figure 1:**
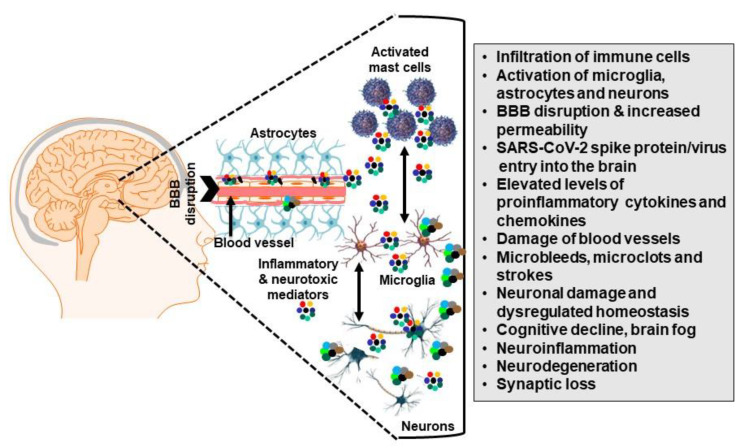
Schematic diagram showing the proposed role of mast cells and microglia in the pathogenesis of neuro-COVID. A variety of triggers including toxins and viruses such as SARS-CoV-2 can reach the hypothalamus, mostly through the nose and olfactory nerve tract. There, they can disrupt the BBB via activation of perivascular mast cells, which then further increase BBB permeability and activate microglia. Proinflammatory molecules released from microglia can damage neurons, disrupt homeostasis, and contribute to the pathogenesis of neuro-COVID.

Mast cells communicate with microglia [32,164], leading to their activation [33,164,165,166] and contributing to neuroinflammation [32,33] and neurodegenerative diseases [32,167]. This effect is not seen in mast-cell-deficient mice [168,169]. In fact, mast cell proteases can trigger astrocytes and glia/neurons and release IL-33 [170]. Stabilization of mast cells was shown to inhibit lipopolysaccharide (LPS)-induced neuroinflammation by suppressing the activation of microglia [171]. Activation of mast cells and microglia in the hypothalamus and brain [172] could lead to cognitive dysfunction [173] and neuronal apoptosis (Figure 1) [173]. In addition, mast cells can activate the hypothalamic–pituitary–adrenal (HPA) axis [174,175,176,177] through the release of histamine [178], IL-6 [179], and CRH [180]. It is interesting that stress has been linked to the possible priming of immune cells thus contributing to neuroinflammation in AD [181,181]. Furthermore, NT [182,183] and substance P (SP) [2] induce CRH receptor-1 (CRHR1) expression in mast cells. Mast-cell-derived histamine [184] and tryptase [185] can trigger microglia and induce neuroinflammation [33]. Mast cells have been shown to be an early activator of LPS-induced neuroinflammation and BBB damage in the hippocampus [172]. In addition, food allergy that depends on mast cell activation has been shown to increase activated microglia and TNF in the hippocampus, associated with behavioral and learning impairments [186]. Another paper reported that early stress in mice and humans disrupted interactions between mast cells and glia via the involvement of histamine [187]. As such, mast cells can participate in neuroinflammation [188,189] by releasing histamine and several inflammatory cytokines and chemokines [190].

## 5. Mast Cells in the CNS

Mast cells are ubiquitous in the body [191]. They are mostly known for mediating allergic and anaphylactic reactions [192], and several other diseases such as mastocytosis [193]. The functions of mast cells in health and several pathologic conditions were reviewed recently [194,195,196,197]. Mast cells respond to allergic but also to various other non-allergic stimuli [193]. Activated mast cells can secrete as many as 100 biologically powerful mediators, including pro-inflammatory molecules [190] such as bradykinin, chymase, histamine, tryptase, chemokine (C-C motif) ligand 2 (CCL2), CXCL8 [198], IL-6 [199], IL-1β, and TNF-α [200]. A particular potent stimulus of the mast cells is the peptide SP, especially when primed by the “alarmin” cytokine IL-33 [201,202,203,204]. In addition, we showed that SP can induce expression of the IL-33 receptor (ST2) [200], thus further increasing mast cell stimulation. Mast cells can also be stimulated to secrete mitochondrial DNA (mtDNA) [205], which serves as an additional “alarmin” and can trigger an auto-inflammatory reaction [206,207]. Mast cells are also found in the CNS perivascularly [29,208], especially in the meninges [28,209] and the median eminence of the hypothalamus [122,209,210], where they could have numerous functions (Table 1). We have called brain mast cells the “immune gate to the brain” [29]. Functional interactions have been reported between mast cells and neurons [209,211] that are often positive for CRH [183,209]. Mast cells are the richest source of histamine in the CNS, particularly in the amygdala, hippocampus, hypothalamus, and thalamus [212,213]. Stimulated brain mast cells contribute to postoperative cognitive dysfunction (POCD) through the release of inflammatory and neurotoxic mediators from activated microglia [86,173]. Activation of mast cells [183] and microglia in the hypothalamus [49] could cause cognitive dysfunction [173] that is also seen in patients with mastocytosis [47,214,215] and may be related to brain abnormalities [216]. Allergic stimulation of nasal mast cells resulted in stimulation of the HPA axis [174,175,176,177], possibly via mast cell release of histamine [178], IL-6 [178,217], and CRH [180]. The influence of stress on mast cells has also been reviewed [140,218]. Restraint stress in rodents increased BBB permeability [210,219,220] via CRH [219,221,222]. Mast-cell-released cytokines [223,224] increased BBB permeability [210,219] and permitted mammary adenocarcinoma brain metastases in mice [221]. This process could worsen with stress, acting via CRH stimulation of mast cells [219,221] and an increase in dura vascular permeability. Meningeal mast cells affected the integrity of the BBB and promoted T-cell brain infiltration [225]. Inflammation mediated by mast cells and microglia disrupted the BBB [226]. Mast cell responsiveness may be regulated not only by the neuroimmune stimuli but also by the effects of the different receptors involved. For instance, mast cells express high-affinity neurokinin-1 (NK-1) receptors for SP [2]. Moreover, SP and NT [182] induced the expression of CRHR-1 in human mast cells. Secretion of mediators can occur by utilizing different signaling [227,228,229,230] and secretory [228,230] pathways. The regulation of mast cells by neurotransmitters and neuropeptides has been reviewed [231,232,233], with emphasis on CRH [177], hemokinin-1 (HK-1) [234], nerve growth factor (NGF [235], NT [236], SP [237], and somatostatin [238,239] acting via activation of high-affinity receptors. Activated mast cells could release a number of pro-inflammatory and vasoactive mediators that could contribute to long COVID syndrome symptoms [177,240]. Some mediators are pre-stored in secretory granules (e.g., histamine, tryptase, and TNF-α) [241,242] and are released immediately following stimulation, while others are newly synthesized and then released, such as chemokines (e.g., CCL2, CCXL8) [198], and cytokines (IL-6 [199], IL-1β [243], TNF-α [200]). Apart from allergic triggers acting via IgE, mast cells are stimulated by non-allergic agents [192,203,244], especially neuropeptides [231], such as SP [237,243] and the SP-related HK-1 [234], which have pro-inflammatory properties. Under such conditions, especially when primed by IL-33 [203,204], mast cells can release various inflammatory mediators without the release of histamine or tryptase [245], thus contributing to inflammatory disorders [189,192]. Moreover, mouse mast-cell proteases 6 (MMCP 6) and MMCP 7 stimulated the release of IL-33 from mouse fetal-brain-derived cultured primary astrocytes in vitro [170]. A case in point is the selective release of IL-6 [199,246], which is elevated in systemic mastocytosis and correlated with disease severity [247,248,249] and can increase mast cell numbers [250].

## 6. Mast Cells in Long COVID

Mast cells are activated by viruses [251,252] such as SARS-CoV-2 [17,18,20,53,55,57,253,254,255,256,257,258,259,260,261]. Recent studies have also reported mast cell activation in the lungs [254] and perivascular inflammation in the brains [75] of COVID-19 patients. We hypothesized that the spike protein can get into the brain either directly or through the activation of mast cells, which then disrupts the integrity of the BBB (Figure 1) [79]. Two studies reported elevated serum levels of chymase in patients with COVID-19 [253,260]. Moreover, a recent study demonstrated that mast cells enhance cellular entry of SARS-CoV-2 through the generation of chymase-spike complexes [52]. Chymase converts angiotensin I to angiotensin II and may act in an autocrine fashion to increase the expression of ACE2, which then facilitate viral entry. Another paper reported that mast-cell-derived histamine can increase SARS-CoV-2 entry into endothelial cells [90]. Mast cells also release extracellular mtDNA [205], which was shown to be significantly elevated in COVID-19 patients [262]. Extracellular mtDNA can then stimulate the secretion of pro-inflammatory mediators from other immunocytes [206,207].

## 7. Neuroimmune Biomarkers

While a number of molecules are elevated in the blood of patients with COVID-19 [34,35,36,263], the results have been inconsistent and have focused primarily on pro-inflammatory mediators. A few studies have investigated blood biomarkers that may reflect brain injury in COVID-19 patients [264,265]. Anti-receptor antibodies and autoimmune gene expression [266] have also been reported. IL-15 is implicated in viral clearance with anti-viral properties, including in COVID-19 [267,268]. We showed elevated IL-18 in the serum of patients with COVID-19 [269]. IL-18 remains elevated longer than other cytokines in inflammatory and autoimmune disorders [270,271], including COVID-19 [269]. Calprotectin (S100A8/A9) was associated with microglia activation [272] and was elevated in the serum of patients with COVID-19 [269]. Calprotectin was also in the CSF of patients with Multiple Sclerosis (MS) [273] and demyelinating polyneuropathy [274]. Neuroligins (NLGs) and neurexins are implicated in synaptic function and cognitive disease [275]. NLG1 levels were reduced in the cortex and the CSF of AD patients [276] or those with mild cognitive impairment (MCI) [277]. NLG4 was associated with cognitive decline [278], while neuropilin-1 (NRP-1) was shown to facilitate SARS-CoV-2 entry by binding to the spike protein [279]. Moreover, S100β was shown to be associated with COVID-19 severity [280] and promote microglia activation [281,282,283] and has been linked to neuroinflammation and cognitive decline [284]. Neurofilament light chain (NfL), microtubule-associated protein-2 (MAP-2), and glial fibrillary acidic protein (GFAP) indicate axonal/neuronal damage and brain injury [264,285,286,287,288]. Elevated levels of osteopontin have been associated with reduced cognition [289,290]. A recent study indicated that COVID-19 was associated with brain pathology in the UK Biobank [291] and was associated with neuroinflammation involving primarily the chemokine CCL11 in a mouse model [292]. CCL11 has been implicated in neuroinflammatory disorders [293], while osteopontin was reported to disrupt the BBB [294]. Chemokine CCL19 and its receptor C-C chemokine receptor type 7 (CCR7) axis are involved in the immune response to viral infections [268,295]. Increased levels of CCL19 were associated with disease severity in COVID-19 patients [296]. 

## 8. Lack of Effective Treatments

**Figure 2:**
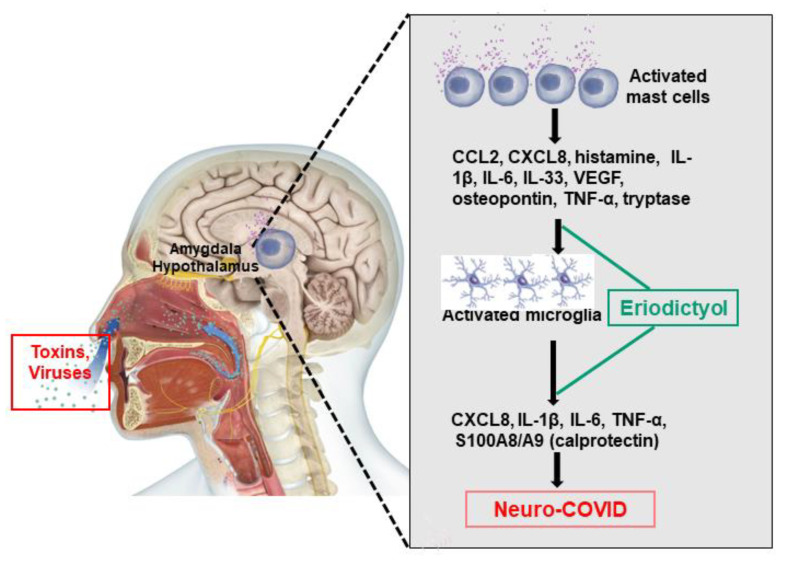
Schematic diagram showing the proposed beneficial effects of eriodictyol. Neuro-COVID can activate mast cells, and several inflammatory mediators released from activated mast cells can activate microglia and other brain cells to release inflammatory and neurotoxic mediators that can cause neuroinflammation and neurodegeneration and exacerbate neuro-COVID disease severity. Eriodictyol could inhibit neuro-COVID-associated mast cell activation-mediated inflammatory mediator release as well as inflammatory mediators released from activated microglia. These inhibitions could reduce the disease severity or treat neuro-COVID.

To date, there are no effective drugs to either treat long COVID or mitigate the release of inflammatory mediators from microglia. Understanding how neuro-immune and toxic triggers contribute to long COVID and how to regulate this response is of clinical importance (Figure 2). One of the major impediments has been the lack of appropriate disease surrogates either in vivo or in vitro [297], as well as the lack of effective inhibitors of neuroinflammation. Apparently, there have been therapeutic considerations of “stabilizing” the BBB [226,298].

For inflammation, non-steroidal anti-inflammatory drugs (NSAIDs) did not improve COVID-19 [297]. Biologics have also been tried in COVID-19. Even though IL-6 has been reported to be elevated and possibly an independent risk factor, clinical trials using IL-6 inhibitors did not show any consistent benefit in COVID-19 [299]. One study reported that a clinically available IL-1β antagonist significantly improved COVID-19 with secondary hemophagocytic lymphohistocytosis (sHLH) that was characterized by pancytopenia, hyper-coagulation, and acute kidney injury [300]. Glucocorticoids have been used extensively in severe, hospitalized patients with COVID-19 [301], but the results are confusing. One paper reported a reduction in mechanical ventilation and a 20 percent reduction in the mortality rate of COVID-19 patients but also longer hospital stays and longer viral clearance time [302]. A more recent systematic review and meta-analysis showed a trend toward a higher discharge rate, but the effect was minimal and not significant [301]. Another analysis of 16 randomized control trials reported that systemic corticosteroids slightly reduced 30-day mortality in severe patients, but there was no benefit up to 120 days [303]. A multicenter observational cohort study conducted in 55 Spanish intensive care units reported that early administration of high doses of dexamethasone since symptom onset could actually prove harmful for 90-day mortality [304]. In fact, it has been argued that even though glucocorticosteroids may improve outcomes in severe, intubated patients with COVID-19, they could also reduce the production of antiviral IgG antibodies [305], thus hampering protection from other infections and worsening long-term outcomes [306].

Inhibition of brain inflammation could instead be accomplished with the use of some natural flavonoids [79,307,308,309,310,311,312]. In particular, the flavone luteolin inhibits both microglia [149,313,314] and mast cells [315,316], as well as related inflammatory processes [147,311]. A novel luteolin analogue, tetramethoxyluteolin [147], can inhibit secretion of the cytokines IL-1β and TNF-α [149], as well as the chemokines CCL2 and CCL5 [198], from human microglia [149,314] and mast cells [220,299]. Flavonoids have been reported to prevent neuroinflammation [311,312,317,318], provide neuroprotection [311,318,319,320,321], and reduce cognitive dysfunction [322,323,324,325,326], especially brain fog [48,327,328]. However, flavonoids are difficult to dissolve in aqueous solutions and also have poor oral absorption and bioavailability. Two formulations containing liposomal luteolin (BrainGain^®^ and FibroProtek^®^ were successfully used to treat a severe COVID-19 patient with brain fog [329]. We have identified a novel flavonoid that is structurally similar to luteolin, the flavanone eriodictyol [330,331,332], which is also partially water-soluble and may be particularly suited for development as an effective treatment (Figure 2) because of its multiple beneficial actions (Table 2) [333,334,335]. A new and novel dietary supplement (ViralProtek^®^) combines eriodictyol [334,335,336,337] with oleuropein from olive leaves [338,339,340] and sulforaphane from broccoli [341] and was recently shown to have strong coronavirus inhibitory properties.

## 9. Conclusions

Neuro-COVID is a common presentation of long COVID patients and could be at least partly caused by the activation of brain mast cells and microglia, leading to perivascular inflammation and disruption of neuronal connectivity and neuronal signal transmission. In the absence of any approved drugs, a combination of certain natural compounds could help minimize these processes and associated symptoms.

## Figures and Tables

**Table 1:** Mast cell actions associated with brain pathophysiology.

Angiogenesis
Activation of microglia, astrocytes, and neurons
Cognitive decline
Disruption of the BBB and entry of peripheral inflammatory mediators, cells, and pathogens/SARS CoV-2 into the brain parenchyma
Early responders in brain injury
Growth factor secretion
Increase vascular permeability
Interactions with microglia, astrocytes, and neurons
Neuroprotection
Neurodegeneration
Neuroinflammation
Proinflammatory mediator release
Posttraumatic stress disorder (PTSD)
Regulation of the HPA axis and stress response
Secretion of proinflammatory, neurotoxic, and vasoactive mediators
Vascular permeability

**Table 2:** Beneficial actions of eriodictyol.

Ameliorates cognitive dysfunctions
ACE2-RBD blocker
Anti-inflammatory
Antioxidant
Cardioprotective
Hepatoprotective
Inhibits brain injury and neurological deficits and improves memory impairment
Inhibits synaptic dysfunctions
Inhibits oxidative stress-associated cell death
Inhibits stress-induced deleterious effects
Neuroprotective
RNA polymerase inhibitor
SARS-CoV-2 protease inhibitor

## Data Availability

Not applicable.

## Abbreviations

AD	Alzheimer’s disease
ACE2	Angiotensin-converting enzyme 2
Apoe4	Apolipoprotein E4
BBB	Blood–brain barrier
CNS	Central nervous system
CSF	Cerebrospinal fluid
CXCL8	Chemokine (C-X-C motif) ligand 8
COVID-19	Coronavirus disease-2019
CRH	Corticotropin-releasing hormone
DAMPs	Damage-associated molecular patterns (DAMPs)
GFAP	Glial fibrillary acidic protein
HK-1	Hemokinin-1
HLA	Human leukocyte antigen
Iba1	Calcium-binding adapter molecule 1
IL-1β	Interleukin-1 beta
LPS	Lipopolysaccharide
MMCP6, 7	Mast cell proteases 6 and 7
MAP-2	Microtubule-associated protein-2
MCI	Mild cognitive impairment
mtDNA	Mitochondrial DNA
MS	Multiple sclerosis
NGF	Nerve growth factor
NfL	Neurofilament light chain
NK-1	Neurokinin-1
NLGs	Neuroligins
NT	Neurotensin
NSAIDs	Non-steroidal anti-inflammatory drugs
NF-κB	Nuclear factor kappa B
NLRP3	Nucleotide-binding domain (NOD)-like receptor protein 3
PAMPs	Pathogen-associated molecular patterns
PD	Parkinson’s disease
PASC	Post-acute sequelae of SARS-CoV-2
PTSD	Post-traumatic stress disorder
S	Spike
SARS-CoV-3	Severe Acute respiratory syndrome coronavirus 2
SP	Substance P
TLRs	Toll-like receptors
TNF	Tumor necrosis factor

## References

[1] Brodin P. (2021). Immune determinants of COVID-19 disease presentation and severity. Nat. Med..

[2] Asadi S., Alysandratos K.D., Angelidou A., Miniati A., Sismanopoulos N., Vasiadi M., Zhang B., Kalogeromitros D., Theoharides T.C. (2012). Substance P (SP) induces expression of functional corticotropin-releasing hormone receptor-1 (CRHR-1) in human mast cells. J. Investig. Dermatol..

[3] Canna S.W., Cron R.Q. (2020). Highways to hell: Mechanism-based management of cytokine storm syndromes. J. Allergy Clin. Immunol..

[4] Giamarellos-Bourboulis E.J., Netea M.G., Rovina N., Akinosoglou K., Antoniadou A., Antonakos N., Damoraki G., Gkavogianni T., Adami M.E., Katsaounou P. (2020). Complex Immune Dysregulation in COVID-19 Patients with Severe Respiratory Failure. Cell Host Microbe.

[5] Ye Q., Wang B., Mao J. (2020). The pathogenesis and treatment of the ‘Cytokine Storm’ in COVID-19. J. Infect..

[6] Chen G., Wu D., Guo W., Cao Y., Huang D., Wang H., Wang T., Zhang X., Chen H., Yu H. (2020). Clinical and immunological features of severe and moderate coronavirus disease 2019. J. Clin. Investig..

[7] Paces J., Strizova Z., Smrz D., Cerny J. (2020). COVID-19 and the immune system. Physiol. Res..

[8] Ragab D., Salah Eldin H., Taeimah M., Khattab R., Salem R. (2020). The COVID-19 Cytokine Storm; What We Know So Far. Front. Immunol..

[9] Copaescu A., Smibert O., Gibson A., Phillips E.J., Trubiano J.A. (2020). The role of IL-6 and other mediators in the cytokine storm associated with SARS-CoV-2 infection. J. Allergy Clin. Immunol..

[10] Moore J.B., June C.H. (2020). Cytokine release syndrome in severe COVID-19. Science.

[11] Herold T., Jurinovic V., Arnreich C., Lipworth B.J., Hellmuth J.C., von Bergwelt-Baildon M., Klein M., Weinberger T. (2020). Elevated levels of IL-6 and CRP predict the need for mechanical ventilation in COVID-19. J. Allergy Clin. Immunol..

[12] Proal A.D., VanElzakker M.B. (2021). Long COVID or Post-acute Sequelae of COVID-19 (PASC): An Overview of Biological Factors That May Contribute to Persistent Symptoms. Front. Microbiol..

[13] Dotan A., Shoenfeld Y. (2022). Post-COVID syndrome: The aftershock of SARS-CoV-2. Int. J. Infect. Dis..

[14] O’Mahoney L.L., Routen A., Gillies C., Ekezie W., Welford A., Zhang A., Karamchandani U., Simms-Williams N., Cassambai S., Ardavani A. (2023). The prevalence and long-term health effects of Long Covid among hospitalised and non-hospitalised populations: A systematic review and meta-analysis. EClinicalMedicine.

[15] Ceban F., Ling S., Lui L.M.W., Lee Y., Gill H., Teopiz K.M., Rodrigues N.B., Subramaniapillai M., Di Vincenzo J.D., Cao B. (2022). Fatigue and cognitive impairment in Post-COVID-19 Syndrome: A systematic review and meta-analysis. Brain Behav. Immun..

[16] Theoharides T.C., Conti P. (2020). COVID-19 and Multisystem Inflammatory Syndrome, or is it Mast Cell Activation Syndrome?. J. Biol. Regul. Homeost. Agents.

[17] Theoharides T.C. (2021). Potential association of mast cells with coronavirus disease 2019. Ann. Allergy Asthma Immunol..

[18] Theoharides T.C. (2020). COVID-19, pulmonary mast cells, cytokine storms, and beneficial actions of luteolin. Biofactors.

[19] Townsend L., Dyer A.H., Jones K., Dunne J., Mooney A., Gaffney F., O’Connor L., Leavy D., O’Brien K., Dowds J. (2020). Persistent fatigue following SARS-CoV-2 infection is common and independent of severity of initial infection. PLoS ONE.

[20] Weinstock L.B., Brook J.B., Walters A.S., Goris A., Afrin L.B., Molderings G.J. (2021). Mast cell activation symptoms are prevalent in Long-COVID. Int. J. Infect. Dis..

[21] Ongur D., Perlis R., Goff D. (2020). Psychiatry and COVID-19. JAMA.

[22] Vindegaard N., Benros M.E. (2020). COVID-19 pandemic and mental health consequences: Systematic review of the current evidence. Brain Behav. Immun..

[23] Pfefferbaum B., North C.S. (2020). Mental Health and the COVID-19 Pandemic. N. Engl. J. Med..

[24] Xiang Y.T., Yang Y., Li W., Zhang L., Zhang Q., Cheung T., Ng C.H. (2020). Timely mental health care for the 2019 novel coronavirus outbreak is urgently needed. Lancet Psychiatry.

[25] Efstathiou V., Stefanou M.I., Demetriou M., Siafakas N., Makris M., Tsivgoulis G., Zoumpourlis V., Kympouropoulos S.P., Tsoporis J.N., Spandidos D.A. (2022). Long COVID and neuropsychiatric manifestations (Review). Exp. Ther. Med..

[26] Badenoch J.B., Rengasamy E.R., Watson C., Jansen K., Chakraborty S., Sundaram R.D., Hafeez D., Burchill E., Saini A., Thomas L. (2022). Persistent neuropsychiatric symptoms after COVID-19: A systematic review and meta-analysis. Brain Commun..

[27] Han Y., Yuan K., Wang Z., Liu W.J., Lu Z.A., Liu L., Shi L., Yan W., Yuan J.L., Li J.L. (2021). Neuropsychiatric manifestations of COVID-19, potential neurotropic mechanisms, and therapeutic interventions. Transl. Psychiatry.

[28] Polyzoidis S., Koletsa T., Panagiotidou S., Ashkan K., Theoharides T.C. (2015). Mast cells in meningiomas and brain inflammation. J. Neuroinflamm..

[29] Theoharides T.C. (1990). Mast cells: The immune gate to the brain. Life Sci..

[30] Zhou Q., Wang Y.W., Ni P.F., Chen Y.N., Dong H.Q., Qian Y.N. (2018). Effect of tryptase on mouse brain microvascular endothelial cells via protease-activated receptor 2. J. Neuroinflamm..

[31] Novak P., Giannetti M.P., Weller E., Hamilton M.J., Castells M. (2022). Mast cell disorders are associated with decreased cerebral blood flow and small fiber neuropathy. Ann. Allergy Asthma Immunol..

[32] Sandhu J.K., Kulka M. (2021). Decoding Mast Cell-Microglia Communication in Neurodegenerative Diseases. Int. J. Mol. Sci..

[33] Skaper S.D., Facci L., Giusti P. (2014). Neuroinflammation, microglia and mast cells in the pathophysiology of neurocognitive disorders: A review. CNS Neurol. Disord. Drug Targets.

[34] Cron R.Q., Goyal G., Chatham W.W. (2022). Cytokine Storm Syndrome. Annu. Rev. Med..

[35] Dutta D., Liu J., Xiong H. (2022). NLRP3 inflammasome activation and SARS-CoV-2-mediated hyperinflammation, cytokine storm and neurological syndromes. Int. J. Physiol. Pathophysiol. Pharmacol..

[36] Rasool G., Riaz M., Abbas M., Fatima H., Qamar M.M., Zafar F., Mahmood Z. (2022). COVID-19: Clinical laboratory diagnosis and monitoring of novel coronavirus infected patients using molecular, serological and biochemical markers: A review. Int. J. Immunopathol. Pharmacol..

[37] (2020). Long COVID: Let patients help define long-lasting COVID symptoms. Nature.

[38] Spudich S., Nath A. (2022). Nervous system consequences of COVID-19. Science.

[39] Finsterer J., Scorza F.A. (2021). Clinical and Pathophysiologic Spectrum of Neuro-COVID. Mol. Neurobiol..

[40] Davies D.A., Adlimoghaddam A., Albensi B.C. (2021). The Effect of COVID-19 on NF-kappaB and Neurological Manifestations of Disease. Mol. Neurobiol..

[41] Norouzi M., Miar P., Norouzi S., Nikpour P. (2021). Nervous System Involvement in COVID-19: A Review of the Current Knowledge. Mol. Neurobiol..

[42] Dewanjee S., Vallamkondu J., Kalra R.S., Puvvada N., Kandimalla R., Reddy P.H. (2021). Emerging COVID-19 Neurological Manifestations: Present Outlook and Potential Neurological Challenges in COVID-19 Pandemic. Mol. Neurobiol..

[43] Patel M.A., Knauer M.J., Nicholson M., Daley M., Van Nynatten L.R., Martin C., Patterson E.K., Cepinskas G., Seney S.L., Dobretzberger V. (2022). Elevated vascular transformation blood biomarkers in Long-COVID indicate angiogenesis as a key pathophysiological mechanism. Mol. Med..

[44] Schou T.M., Joca S., Wegener G., Bay-Richter C. (2021). Psychiatric and neuropsychiatric sequelae of COVID-19—A systematic review. Brain Behav. Immun..

[45] Vikse J., Omdal R. (2019). Fatigue in Mastocytosis: A Case Series. Clin. Ther..

[46] Georgin-Lavialle S., Gaillard R., Moura D., Hermine O. (2016). Mastocytosis in adulthood and neuropsychiatric disorders. Transl. Res..

[47] Afrin L.B., Pohlau D., Raithel M., Haenisch B., Dumoulin F.L., Homann J., Mauer U.M., Harzer S., Molderings G.J. (2015). Mast cell activation disease: An underappreciated cause of neurologic and psychiatric symptoms and diseases. Brain Behav. Immun..

[48] Theoharides T.C., Cholevas C., Polyzoidis K., Politis A. (2021). Long-COVID syndrome-associated brain fog and chemofog: Luteolin to the rescue. Biofactors.

[49] Hatziagelaki E., Adamaki M., Tsilioni I., Dimitriadis G., Theoharides T.C. (2018). Myalgic Encephalomyelitis/Chronic Fatigue Syndrome-Metabolic Disease or Disturbed Homeostasis due to Focal Inflammation in the Hypothalamus?. J. Pharmacol. Exp. Ther..

[50] Sukocheva O.A., Maksoud R., Beeraka N.M., Madhunapantula S.V., Sinelnikov M., Nikolenko V.N., Neganova M.E., Klochkov S.G., Amjad Kamal M., Staines D.R. (2022). Analysis of post COVID-19 condition and its overlap with myalgic encephalomyelitis/chronic fatigue syndrome. J. Adv. Res..

[51] Kempuraj D., Selvakumar G.P., Thangavel R., Ahmed M.E., Zaheer S., Kumar K.K., Yelam A., Kaur H., Dubova I., Raikwar S.P. (2018). Glia Maturation Factor and Mast Cell-Dependent Expression of Inflammatory Mediators and Proteinase Activated Receptor-2 in Neuroinflammation. J. Alzheimers Dis..

[52] Liu S., Suzuki Y., Takemasa E., Watanabe R., Mogi M. (2022). Mast cells promote viral entry of SARS-CoV-2 via formation of chymase/spike protein complex. Eur. J. Pharmacol..

[53] Arun S., Storan A., Myers B. (2022). Mast cell activation syndrome and the link with long COVID. Br. J. Hosp. Med..

[54] Murdaca G., Di Gioacchino M., Greco M., Borro M., Paladin F., Petrarca C., Gangemi S. (2021). Basophils and Mast Cells in COVID-19 Pathogenesis. Cells.

[55] Afrin L.B., Weinstock L.B., Molderings G.J. (2020). COVID-19 hyperinflammation and post-COVID-19 illness may be rooted in mast cell activation syndrome. Int. J. Infect. Dis..

[56] Ozdemir O., Goksu Erol A.Y., Dikici U. (2022). Mast Cell’s Role in Cytokine Release Syndrome and Related Manifestations of COVID-19 Disease. Curr. Pharm. Des..

[57] Hafezi B., Chan L., Knapp J.P., Karimi N., Alizadeh K., Mehrani Y., Bridle B.W., Karimi K. (2021). Cytokine Storm Syndrome in SARS-CoV-2 Infections: A Functional Role of Mast Cells. Cells.

[58] Phillips S., Williams M.A. (2021). Confronting Our Next National Health Disaster—Long-Haul Covid. N. Engl. J. Med..

[59] Iacurci G. (2022). Your Health, Your Money. Long Covid May Be “The Next Public Health Disaster”—With a $3.7 Trillion Economic Impact Rivaline the Great Recession.

[60] Tremblay M.E., Madore C., Bordeleau M., Tian L., Verkhratsky A. (2020). Neuropathobiology of COVID-19: The Role for Glia. Front. Cell. Neurosci..

[61] McMahon C.L., Staples H., Gazi M., Carrion R., Hsieh J. (2021). SARS-CoV-2 targets glial cells in human cortical organoids. Stem Cell Rep..

[62] Butowt R., von Bartheld C.S. (2022). The route of SARS-CoV-2 to brain infection: Have we been barking up the wrong tree?. Mol. Neurodegener..

[63] Tai W., He L., Zhang X., Pu J., Voronin D., Jiang S., Zhou Y., Du L. (2020). Characterization of the receptor-binding domain (RBD) of 2019 novel coronavirus: Implication for development of RBD protein as a viral attachment inhibitor and vaccine. Cell Mol. Immunol..

[64] Espindola O.M., Gomes Y.C.P., Brandao C.O., Torres R.C., Siqueira M., Soares C.N., Lima M., Leite A., Venturotti C.O., Carvalho A.J.C. (2021). Inflammatory Cytokine Patterns Associated with Neurological Diseases in Coronavirus Disease 2019. Ann. Neurol..

[65] Oka Y., Ueda A., Nakagawa T., Kikuchi Y., Inoue D., Marumo S., Matsumoto S. (2021). SARS-CoV-2-related Progressive Brain White Matter Lesion Associated with an Increased Cerebrospinal Fluid Level of IL-6. Intern. Med..

[66] Meinhardt J., Radke J., Dittmayer C., Franz J., Thomas C., Mothes R., Laue M., Schneider J., Brunink S., Greuel S. (2021). Olfactory transmucosal SARS-CoV-2 invasion as a port of central nervous system entry in individuals with COVID-19. Nat. Neurosci..

[67] Jiao L., Yang Y., Yu W., Zhao Y., Long H., Gao J., Ding K., Ma C., Li J., Zhao S. (2021). The olfactory route is a potential way for SARS-CoV-2 to invade the central nervous system of rhesus monkeys. Signal Transduct. Target. Ther..

[68] Gomes I., Karmirian K., Oliveira J.T., Pedrosa C., Mendes M.A., Rosman F.C., Chimelli L., Rehen S. (2021). SARS-CoV-2 infection of the central nervous system in a 14-month-old child: A case report of a complete autopsy. Lancet Reg. Health Am..

[69] Yang A.C., Kern F., Losada P.M., Agam M.R., Maat C.A., Schmartz G.P., Fehlmann T., Stein J.A., Schaum N., Lee D.P. (2021). Dysregulation of brain and choroid plexus cell types in severe COVID-19. Nature.

[70] Brann D.H., Tsukahara T., Weinreb C., Lipovsek M., Van den Berge K., Gong B., Chance R., Macaulay I.C., Chou H.J., Fletcher R.B. (2020). Non-neuronal expression of SARS-CoV-2 entry genes in the olfactory system suggests mechanisms underlying COVID-19-associated anosmia. Sci. Adv..

[71] Xu J., Zhong S., Liu J., Li L., Li Y., Wu X., Li Z., Deng P., Zhang J., Zhong N. (2005). Detection of severe acute respiratory syndrome coronavirus in the brain: Potential role of the chemokine mig in pathogenesis. Clin. Infect. Dis..

[72] Karnik M., Beeraka N.M., Uthaiah C.A., Nataraj S.M., Bettadapura A.D.S., Aliev G., Madhunapantula S.V. (2021). A Review on SARS-CoV-2-Induced Neuroinflammation, Neurodevelopmental Complications, and Recent Updates on the Vaccine Development. Mol. Neurobiol..

[73] Sodagar A., Javed R., Tahir H., Razak S.I.A., Shakir M., Naeem M., Yusof A.H.A., Sagadevan S., Hazafa A., Uddin J. (2022). Pathological Features and Neuroinflammatory Mechanisms of SARS-CoV-2 in the Brain and Potential Therapeutic Approaches. Biomolecules.

[74] Tremblay M.E., Madore C., Tian L., Verkhratsky A. (2022). Editorial: Role of Neuroinflammation in the Neuropsychiatric and Neurological Aspects of COVID-19. Front. Cell. Neurosci..

[75] Lee M.H., Perl D.P., Nair G., Li W., Maric D., Murray H., Dodd S.J., Koretsky A.P., Watts J.A., Cheung V. (2021). Microvascular Injury in the Brains of Patients with COVID-19. N. Engl. J. Med..

[76] Adesse D., Gladulich L., Alvarez-Rosa L., Siqueira M., Marcos A.C., Heider M., Motta C.S., Torices S., Toborek M., Stipursky J. (2022). Role of aging in Blood-Brain Barrier dysfunction and susceptibility to SARS-CoV-2 infection: Impacts on neurological symptoms of COVID-19. Fluids Barriers CNS.

[77] Bodnar B., Patel K., Ho W., Luo J.J., Hu W. (2021). Cellular mechanisms underlying neurological/neuropsychiatric manifestations of COVID-19. J. Med. Virol..

[78] Ng J.H., Sun A., Je H.S., Tan E.K. (2021). Unravelling Pathophysiology of Neurological and Psychiatric Complications of COVID-19 Using Brain Organoids. Neuroscientist.

[79] Theoharides T.C. (2022). Could SARS-CoV-2 Spike Protein Be Responsible for Long-COVID Syndrome?. Mol. Neurobiol..

[80] Song E., Zhang C., Israelow B., Lu-Culligan A., Prado A.V., Skriabine S., Lu P., Weizman O.E., Liu F., Dai Y. (2021). Neuroinvasion of SARS-CoV-2 in human and mouse brain. J. Exp. Med..

[81] Veleri S. (2022). Neurotropism of SARS-CoV-2 and neurological diseases of the central nervous system in COVID-19 patients. Exp. Brain Res..

[82] Cai Y., Zhang J., Xiao T., Peng H., Sterling S.M., Walsh R.M., Rawson S., Rits-Volloch S., Chen B. (2020). Distinct conformational states of SARS-CoV-2 spike protein. Science.

[83] Jeong G.U., Lyu J., Kim K.D., Chung Y.C., Yoon G.Y., Lee S., Hwang I., Shin W.H., Ko J., Lee J.Y. (2022). SARS-CoV-2 Infection of Microglia Elicits Proinflammatory Activation and Apoptotic Cell Death. Microbiol. Spectr..

[84] Olajide O.A., Iwuanyanwu V.U., Adegbola O.D., Al-Hindawi A.A. (2022). SARS-CoV-2 Spike Glycoprotein S1 Induces Neuroinflammation in BV-2 Microglia. Mol. Neurobiol..

[85] Samudyata, Oliveira A.O., Malwade S., Rufino de Sousa N., Goparaju S.K., Gracias J., Orhan F., Steponaviciute L., Schalling M., Sheridan S.D. (2022). SARS-CoV-2 promotes microglial synapse elimination in human brain organoids. Mol. Psychiatry.

[86] Frank M.G., Nguyen K.H., Ball J.B., Hopkins S., Kelley T., Baratta M.V., Fleshner M., Maier S.F. (2022). SARS-CoV-2 spike S1 subunit induces neuroinflammatory, microglial and behavioral sickness responses: Evidence of PAMP-like properties. Brain Behav. Immun..

[87] Savelieff M.G., Feldman E.L., Stino A.M. (2022). Neurological sequela and disruption of neuron-glia homeostasis in SARS-CoV-2 infection. Neurobiol. Dis..

[88] Kim E.S., Jeon M.T., Kim K.S., Lee S., Kim S., Kim D.G. (2021). Spike Proteins of SARS-CoV-2 Induce Pathological Changes in Molecular Delivery and Metabolic Function in the Brain Endothelial Cells. Viruses.

[89] Perico L., Morigi M., Galbusera M., Pezzotta A., Gastoldi S., Imberti B., Perna A., Ruggenenti P., Donadelli R., Benigni A. (2022). SARS-CoV-2 Spike Protein 1 Activates Microvascular Endothelial Cells and Complement System Leading to Platelet Aggregation. Front. Immunol..

[90] Raghavan S., Leo M.D. (2022). Histamine Potentiates SARS-CoV-2 Spike Protein Entry Into Endothelial Cells. Front. Pharmacol..

[91] Yang R.C., Huang K., Zhang H.P., Li L., Zhang Y.F., Tan C., Chen H.C., Jin M.L., Wang X.R. (2022). SARS-CoV-2 productively infects human brain microvascular endothelial cells. J. Neuroinflamm..

[92] Erickson M.A., Rhea E.M., Knopp R.C., Banks W.A. (2021). Interactions of SARS-CoV-2 with the Blood-Brain Barrier. Int. J. Mol. Sci..

[93] Buzhdygan T.P., DeOre B.J., Baldwin-Leclair A., Bullock T.A., McGary H.M., Khan J.A., Razmpour R., Hale J.F., Galie P.A., Potula R. (2020). The SARS-CoV-2 spike protein alters barrier function in 2D static and 3D microfluidic in-vitro models of the human blood-brain barrier. Neurobiol. Dis..

[94] DeOre B.J., Tran K.A., Andrews A.M., Ramirez S.H., Galie P.A. (2021). SARS-CoV-2 Spike Protein Disrupts Blood-Brain Barrier Integrity via RhoA Activation. J. Neuroimmune Pharmacol..

[95] Nation D.A., Sweeney M.D., Montagne A., Sagare A.P., D’Orazio L.M., Pachicano M., Sepehrband F., Nelson A.R., Buennagel D.P., Harrington M.G. (2019). Blood-brain barrier breakdown is an early biomarker of human cognitive dysfunction. Nat. Med..

[96] Zhang L., Zhou L., Bao L., Liu J., Zhu H., Lv Q., Liu R., Chen W., Tong W., Wei Q. (2021). SARS-CoV-2 crosses the blood-brain barrier accompanied with basement membrane disruption without tight junctions alteration. Signal Transduct. Target. Ther..

[97] Krasemann S., Haferkamp U., Pfefferle S., Woo M.S., Heinrich F., Schweizer M., Appelt-Menzel A., Cubukova A., Barenberg J., Leu J. (2022). The blood-brain barrier is dysregulated in COVID-19 and serves as a CNS entry route for SARS-CoV-2. Stem Cell Rep..

[98] Petrovszki D., Walter F.R., Vigh J.P., Kocsis A., Valkai S., Deli M.A., Der A. (2022). Penetration of the SARS-CoV-2 Spike Protein across the Blood-Brain Barrier, as Revealed by a Combination of a Human Cell Culture Model System and Optical Biosensing. Biomedicines.

[99] Wirth K.J., Scheibenbogen C., Paul F. (2021). An attempt to explain the neurological symptoms of Myalgic Encephalomyelitis/Chronic Fatigue Syndrome. J. Transl. Med..

[100] Sun Z., Zhao H., Fang D., Davis C.T., Shi D.S., Lei K., Rich B.E., Winter J.M., Guo L., Sorensen L.K. (2022). Neuroinflammatory disease disrupts the blood-CNS barrier via crosstalk between proinflammatory and endothelial-to-mesenchymal-transition signaling. Neuron.

[101] Wierzba-Bobrowicz T., Krajewski P., Tarka S., Acewicz A., Felczak P., Stepien T., Golan M.P., Grzegorczyk M. (2021). Neuropathological analysis of the brains of fifty-two patients with COVID-19. Folia Neuropathol..

[102] Etter M.M., Martins T.A., Kulsvehagen L., Possnecker E., Duchemin W., Hogan S., Sanabria-Diaz G., Muller J., Chiappini A., Rychen J. (2022). Severe Neuro-COVID is associated with peripheral immune signatures, autoimmunity and neurodegeneration: A prospective cross-sectional study. Nat. Commun..

[103] Chaumont H., Kaczorowski F., San-Galli A., Michel P.P., Tressieres B., Roze E., Quadrio I., Lannuzel A. (2022). Cerebrospinal fluid biomarkers in SARS-CoV-2 patients with acute neurological syndromes. Rev. Neurol..

[104] Ferreira de Araujo J.L., Menezes D., Saraiva-Duarte J.M., de Lima Ferreira L., Santana de Aguiar R., Pedra de Souza R. (2022). Systematic review of host genetic association with COVID-19 prognosis and susceptibility: What have we learned in 2020?. Rev. Med. Virol..

[105] Gkouskou K., Vasilogiannakopoulou T., Andreakos E., Davanos N., Gazouli M., Sanoudou D., Eliopoulos A.G. (2021). COVID-19 enters the expanding network of apolipoprotein E4-related pathologies. Redox. Biol..

[106] Ortiz G.G., Velazquez-Brizuela I.E., Ortiz-Velazquez G.E., Ocampo-Alfaro M.J., Salazar-Flores J., Delgado-Lara D.L.C., Torres-Sanchez E.D. (2022). Alzheimer’s Disease and SARS-CoV-2: Pathophysiological Analysis and Social Context. Brain Sci..

[107] Goldstein M.R., Poland G.A., Graeber A.C.W. (2020). Does apolipoprotein E genotype predict COVID-19 severity?. QJM.

[108] Gupta K., Kaur G., Pathak T., Banerjee I. (2022). Systematic review and meta-analysis of human genetic variants contributing to COVID-19 susceptibility and severity. Gene.

[109] Montagne A., Nation D.A., Sagare A.P., Barisano G., Sweeney M.D., Chakhoyan A., Pachicano M., Joe E., Nelson A.R., D’Orazio L.M. (2020). APOE4 leads to blood-brain barrier dysfunction predicting cognitive decline. Nature.

[110] Onofrio L., Caraglia M., Facchini G., Margherita V., Placido S., Buonerba C. (2020). Toll-like receptors and COVID-19: A two-faced story with an exciting ending. Future Sci. OA.

[111] Sariol A., Perlman S. (2021). SARS-CoV-2 takes its Toll. Nat. Immunol..

[112] Singh H., Singh A., Khan A.A., Gupta V. (2021). Immune mediating molecules and pathogenesis of COVID-19-associated neurological disease. Microb. Pathog..

[113] Khanmohammadi S., Rezaei N. (2021). Role of Toll-like receptors in the pathogenesis of COVID-19. J. Med. Virol..

[114] Jack C.S., Arbour N., Manusow J., Montgrain V., Blain M., McCrea E., Shapiro A., Antel J.P. (2005). TLR signaling tailors innate immune responses in human microglia and astrocytes. J. Immunol..

[115] Vargas G., Medeiros Geraldo L.H., Gedeao Salomao N., Viana Paes M., Regina Souza Lima F., Carvalho Alcantara Gomes F. (2020). Severe acute respiratory syndrome coronavirus 2 (SARS-CoV-2) and glial cells: Insights and perspectives. Brain Behav. Immun. Health.

[116] Zheng M., Karki R., Williams E.P., Yang D., Fitzpatrick E., Vogel P., Jonsson C.B., Kanneganti T.D. (2021). TLR2 senses the SARS-CoV-2 envelope protein to produce inflammatory cytokines. Nat. Immunol..

[117] Aboudounya M.M., Heads R.J. (2021). COVID-19 and Toll-Like Receptor 4 (TLR4): SARS-CoV-2 May Bind and Activate TLR4 to Increase ACE2 Expression, Facilitating Entry and Causing Hyperinflammation. Mediat. Inflamm..

[118] Manan A., Pirzada R.H., Haseeb M., Choi S. (2022). Toll-like Receptor Mediation in SARS-CoV-2: A Therapeutic Approach. Int. J. Mol. Sci..

[119] Kaushik D., Bhandari R., Kuhad A. (2021). TLR4 as a therapeutic target for respiratory and neurological complications of SARS-CoV-2. Expert Opin. Ther. Targets.

[120] Tsilioni I., Theoharides T.C. (2023). Recombinant SARS-CoV-2 Spike Protein and its Receptor Binding Domain stimulate release of different pro-inflammatory mediators via activation of distinct receptors on human microglia cells. Res. Sq..

[121] Dos Santos S.E., Medeiros M., Porfirio J., Tavares W., Pessoa L., Grinberg L., Leite R.E.P., Ferretti-Rebustini R.E.L., Suemoto C.K., Filho W.J. (2020). Similar Microglial Cell Densities across Brain Structures and Mammalian Species: Implications for Brain Tissue Function. J. Neurosci..

[122] Colonna M., Butovsky O. (2017). Microglia Function in the Central Nervous System During Health and Neurodegeneration. Annu. Rev. Immunol..

[123] Perry V.H., Nicoll J.A., Holmes C. (2010). Microglia in neurodegenerative disease. Nat. Rev. Neurol..

[124] Ransohoff R.M. (2016). How neuroinflammation contributes to neurodegeneration. Science.

[125] Angelova D.M., Brown D.R. (2019). Microglia and the aging brain: Are senescent microglia the key to neurodegeneration?. J. Neurochem..

[126] Bachiller S., Jimenez-Ferrer I., Paulus A., Yang Y., Swanberg M., Deierborg T., Boza-Serrano A. (2018). Microglia in Neurological Diseases: A Road Map to Brain-Disease Dependent-Inflammatory Response. Front. Cell. Neurosci..

[127] Hickman S., Izzy S., Sen P., Morsett L., El K.J. (2018). Microglia in neurodegeneration. Nat. Neurosci..

[128] Liberman A.C., Trias E., da Silva C.L., Trindade P., Dos Santos P.M., Refojo D., Hedin-Pereira C., Serfaty C.A. (2018). Neuroimmune and Inflammatory Signals in Complex Disorders of the Central Nervous System. Neuroimmunomodulation.

[129] Xu L., He D., Bai Y. (2016). Microglia-Mediated Inflammation and Neurodegenerative Disease. Mol. Neurobiol..

[130] Subhramanyam C.S., Wang C., Hu Q., Dheen S.T. (2019). Microglia-mediated neuroinflammation in neurodegenerative diseases. Semin. Cell Dev. Biol..

[131] Voet S., Prinz M., van Loo G. (2019). Microglia in Central Nervous System Inflammation and Multiple Sclerosis Pathology. Trends Mol. Med..

[132] Goncalves de Andrade E., Simoncicova E., Carrier M., Vecchiarelli H.A., Robert M.E., Tremblay M.E. (2021). Microglia Fighting for Neurological and Mental Health: On the Central Nervous System Frontline of COVID-19 Pandemic. Front. Cell. Neurosci..

[133] Schwabenland M., Salie H., Tanevski J., Killmer S., Lago M.S., Schlaak A.E., Mayer L., Matschke J., Puschel K., Fitzek A. (2021). Deep spatial profiling of human COVID-19 brains reveals neuroinflammation with distinct microanatomical microglia-T-cell interactions. Immunity.

[134] Poloni T.E., Medici V., Moretti M., Visona S.D., Cirrincione A., Carlos A.F., Davin A., Gagliardi S., Pansarasa O., Cereda C. (2021). COVID-19-related neuropathology and microglial activation in elderly with and without dementia. Brain Pathol..

[135] Poloni T.E., Moretti M., Medici V., Turturici E., Belli G., Cavriani E., Visona S.D., Rossi M., Fantini V., Ferrari R.R. (2022). COVID-19 Pathology in the Lung, Kidney, Heart and Brain: The Different Roles of T-Cells, Macrophages, and Microthrombosis. Cells.

[136] Rai S.N., Tiwari N., Singh P., Singh A.K., Mishra D., Imran M., Singh S., Hooshmandi E., Vamanu E., Singh S.K. (2022). Exploring the Paradox of COVID-19 in Neurological Complications with Emphasis on Parkinson’s and Alzheimer’s Disease. Oxid. Med. Cell. Longev..

[137] Baazaoui N., Iqbal K. (2022). COVID-19 and Neurodegenerative Diseases: Prion-Like Spread and Long-Term Consequences. J. Alzheimers Dis..

[138] Fu Y.W., Xu H.S., Liu S.J. (2022). COVID-19 and neurodegenerative diseases. Eur. Rev. Med. Pharmacol. Sci..

[139] Steenblock C., Todorov V., Kanczkowski W., Eisenhofer G., Schedl A., Wong M.L., Licinio J., Bauer M., Young A.H., Gainetdinov R.R. (2020). Severe acute respiratory syndrome coronavirus 2 (SARS-CoV-2) and the neuroendocrine stress axis. Mol. Psychiatry.

[140] Theoharides T.C. (2020). The impact of psychological stress on mast cells. Ann. Allergy Asthma Immunol..

[141] Manchia M., Gathier A.W., Yapici-Eser H., Schmidt M.V., de Quervain D., van Amelsvoort T., Bisson J.I., Cryan J.F., Howes O.D., Pinto L. (2022). The impact of the prolonged COVID-19 pandemic on stress resilience and mental health: A critical review across waves. Eur. Neuropsychopharmacol..

[142] Lindert J., Jakubauskiene M., Bilsen J. (2021). The COVID-19 disaster and mental health-assessing, responding and recovering. Eur. J. Public Health.

[143] Dijkstra A., Elbert S.P. (2020). Detecting and Preventing Defensive Reactions Toward Persuasive Information on Fruit and Vegetable Consumption Using Induced Eye Movements. Front. Psychol..

[144] Patra R., Das N.C., Mukherjee S. (2021). Toll-Like Receptors (TLRs) as Therapeutic Targets for Treating SARS-CoV-2: An Immunobiological Perspective. Adv. Exp. Med. Biol..

[145] Wallach T., Raden M., Hinkelmann L., Brehm M., Rabsch D., Weidling H., Kruger C., Kettenmann H., Backofen R., Lehnardt S. (2022). Distinct SARS-CoV-2 RNA fragments activate Toll-like receptors 7 and 8 and induce cytokine release from human macrophages and microglia. Front. Immunol..

[146] Martin S., Dicou E., Vincent J.P., Mazella J. (2005). Neurotensin and the neurotensin receptor-3 in microglial cells. J. Neurosci. Res..

[147] Wang W., Ji P., Riopelle R.J., Dow K.E. (2002). Functional expression of corticotropin-releasing hormone (CRH) receptor 1 in cultured rat microglia. J. Neurochem..

[148] Kempuraj D., Selvakumar G.P., Ahmed M.E., Raikwar S.P., Thangavel R., Khan A., Zaheer S.A., Iyer S.S., Burton C., James D. (2020). COVID-19, Mast Cells, Cytokine Storm, Psychological Stress, and Neuroinflammation. Neuroscientist.

[149] Patel A.B., Tsilioni I., Leeman S.E., Theoharides T.C. (2016). Neurotensin stimulates sortilin and mTOR in human microglia inhibitable by methoxyluteolin, a potential therapeutic target for autism. Proc. Natl. Acad. Sci. USA.

[150] Niraula A., Sheridan J.F., Godbout J.P. (2016). Microglia Priming with Aging and Stress. Neuropsychopharmacology.

[151] Carrier M., Simoncicova E., St-Pierre M.K., McKee C., Tremblay M.E. (2021). Psychological Stress as a Risk Factor for Accelerated Cellular Aging and Cognitive Decline: The Involvement of Microglia-Neuron Crosstalk. Front. Mol. Neurosci..

[152] Rahimian R., Wakid M., O’Leary L.A., Mechawar N. (2021). The emerging tale of microglia in psychiatric disorders. Neurosci. Biobehav. Rev..

[153] Wohleb E.S. (2016). Neuron-Microglia Interactions in Mental Health Disorders: “For Better, and For Worse”. Front. Immunol..

[154] Troubat R., Barone P., Leman S., Desmidt T., Cressant A., Atanasova B., Brizard B., El Hage W., Surget A., Belzung C. (2021). Neuroinflammation and depression: A review. Eur. J. Neurosci..

[155] Brites D., Fernandes A. (2015). Neuroinflammation and Depression: Microglia Activation, Extracellular Microvesicles and microRNA Dysregulation. Front. Cell. Neurosci..

[156] Steardo L., Steardo L., Verkhratsky A. (2020). Psychiatric face of COVID-19. Transl. Psychiatry.

[157] Dixon L., Varley J., Gontsarova A., Mallon D., Tona F., Muir D., Luqmani A., Jenkins I.H., Nicholas R., Jones B. (2020). COVID-19-related acute necrotizing encephalopathy with brain stem involvement in a patient with aplastic anemia. Neurol. Neuroimmunol. Neuroinflamm..

[158] Boroujeni M.E., Simani L., Bluyssen H.A.R., Samadikhah H.R., Zamanlui Benisi S., Hassani S., Akbari Dilmaghani N., Fathi M., Vakili K., Mahmoudiasl G.R. (2021). Inflammatory Response Leads to Neuronal Death in Human Post-Mortem Cerebral Cortex in Patients with COVID-19. ACS Chem. Neurosci..

[159] Shen W.B., Logue J., Yang P., Baracco L., Elahi M., Reece E.A., Wang B., Li L., Blanchard T.G., Han Z. (2022). SARS-CoV-2 invades cognitive centers of the brain and induces Alzheimer’s-like neuropathology. bioRxiv.

[160] Radhakrishnan R.K., Kandasamy M. (2022). SARS-CoV-2-Mediated Neuropathogenesis, Deterioration of Hippocampal Neurogenesis and Dementia. Am. J. Alzheimers Dis. Other Demen..

[161] Welcome M.O., Mastorakis N.E. (2021). Neuropathophysiology of coronavirus disease 2019: Neuroinflammation and blood brain barrier disruption are critical pathophysiological processes that contribute to the clinical symptoms of SARS-CoV-2 infection. Inflammopharmacology.

[162] Magro C.M., Mulvey J., Kubiak J., Mikhail S., Suster D., Crowson A.N., Laurence J., Nuovo G. (2021). Severe COVID-19: A multifaceted viral vasculopathy syndrome. Ann. Diagn. Pathol..

[163] Harvey W.T., Carabelli A.M., Jackson B., Gupta R.K., Thomson E.C., Harrison E.M., Ludden C., Reeve R., Rambaut A., Consortium C.-G.U. (2021). SARS-CoV-2 variants, spike mutations and immune escape. Nat. Rev. Microbiol..

[164] Hendriksen E., van Bergeijk D., Oosting R.S., Redegeld F.A. (2017). Mast cells in neuroinflammation and brain disorders. Neurosci. Biobehav. Rev..

[165] Skaper S.D., Facci L., Zusso M., Giusti P. (2017). Neuroinflammation, Mast Cells, and Glia: Dangerous Liaisons. Neuroscientist.

[166] Zhang X., Wang Y., Dong H., Xu Y., Zhang S. (2016). Induction of Microglial Activation by Mediators Released from Mast Cells. Cell. Physiol. Biochem..

[167] Kempuraj D., Selvakumar G.P., Zaheer S., Thangavel R., Ahmed M.E., Raikwar S., Govindarajan R., Iyer S., Zaheer A. (2018). Cross-Talk between Glia, Neurons and Mast Cells in Neuroinflammation Associated with Parkinson’s Disease. J. Neuroimmune Pharmacol..

[168] Dong H., Zhang X., Wang Y., Zhou X., Qian Y., Zhang S. (2017). Suppression of Brain Mast Cells Degranulation Inhibits Microglial Activation and Central Nervous System Inflammation. Mol. Neurobiol..

[169] Selvakumar G.P., Ahmed M.E., Thangavel R., Kempuraj D., Dubova I., Raikwar S.P., Zaheer S., Iyer S.S., Zaheer A. (2020). A role for glia maturation factor dependent activation of mast cells and microglia in MPTP induced dopamine loss and behavioural deficits in mice. Brain Behav. Immun..

[170] Kempuraj D., Thangavel R., Selvakumar G.P., Ahmed M.E., Zaheer S., Raikwar S.P., Zahoor H., Saeed D., Dubova I., Giler G. (2019). Mast Cell Proteases Activate Astrocytes and Glia-Neurons and Release Interleukin-33 by Activating p38 and ERK1/2 MAPKs and NF-kappaB. Mol. Neurobiol..

[171] Dong H., Wang Y., Zhang X., Zhang X., Qian Y., Ding H., Zhang S. (2019). Stabilization of Brain Mast Cells Alleviates LPS-Induced Neuroinflammation by Inhibiting Microglia Activation. Front. Cell. Neurosci..

[172] Wang Y., Sha H., Zhou L., Chen Y., Zhou Q., Dong H., Qian Y. (2020). The Mast Cell Is an Early Activator of Lipopolysaccharide-Induced Neuroinflammation and Blood-Brain Barrier Dysfunction in the Hippocampus. Mediat. Inflamm..

[173] Zhang X., Dong H., Li N., Zhang S., Sun J., Zhang S., Qian Y. (2016). Activated brain mast cells contribute to postoperative cognitive dysfunction by evoking microglia activation and neuronal apoptosis 1. J. Neuroinflamm..

[174] Bugajski A.J., Chlap Z., Gadek-Michalska A., Borycz J., Bugajski J. (1995). Degranulation and decrease in histamine levels of thalamic mast cells coincides with corticosterone secretion induced by compound 48/80. Inflamm. Res..

[175] Kalogeromitros D., Syrigou E.I., Makris M., Kempuraj D., Stavrianeas N.G., Vasiadi M., Theoharides T.C. (2007). Nasal provocation of patients with allergic rhinitis and the hypothalamic-pituitary-adrenal axis. Ann. Allergy Asthma Immunol..

[176] Matsumoto I., Inoue Y., Shimada T., Aikawa T. (2001). Brain mast cells act as an immune gate to the hypothalamic-pituitary-adrenal axis in dogs. J. Exp. Med..

[177] Theoharides T.C., Donelan J.M., Papadopoulou N., Cao J., Kempuraj D., Conti P. (2004). Mast cells as targets of corticotropin-releasing factor and related peptides. Trends Pharmacol. Sci..

[178] Scaccianoce S., Lombardo K., Nicolai R., Affricano D., Angelucci L. (2000). Studies on the involvement of histamine in the hypothalamic-pituitary-adrenal axis activation induced by nerve growth factor. Life Sci..

[179] Mastorakos G., Chrousos G.P., Weber J.S. (1993). Recombinant interleukin-6 activates the hypothalamic-pituitary-adrenal axis in humans. J. Clin. Endocrinol. Metab..

[180] Kempuraj D., Papadopoulou N.G., Lytinas M., Huang M., Kandere-Grzybowska K., Madhappan B., Boucher W., Christodoulou S., Athanassiou A., Theoharides T.C. (2004). Corticotropin-releasing hormone and its structurally related urocortin are synthesized and secreted by human mast cells. Endocrinology.

[181] Milligan A.A., Porter T., Quek H., White A., Haynes J., Jackaman C., Villemagne V., Munyard K., Laws S.M., Verdile G. (2021). Chronic stress and Alzheimer’s disease: The interplay between the hypothalamic-pituitary-adrenal axis, genetics and microglia. Biol. Rev. Camb. Philos. Soc..

[182] Alysandratos K.D., Asadi S., Angelidou A., Zhang B., Sismanopoulos N., Yang H., Critchfield A., Theoharides T.C. (2012). Neurotensin and CRH interactions augment human mast cell activation. PLoS ONE.

[183] Kempuraj D., Mentor S., Thangavel R., Ahmed M.E., Selvakumar G.P., Raikwar S.P., Dubova I., Zaheer S., Iyer S.S., Zaheer A. (2019). Mast Cells in Stress, Pain, Blood-Brain Barrier, Neuroinflammation and Alzheimer’s Disease. Front. Cell. Neurosci..

[184] Zhang W., Zhang X., Zhang Y., Qu C., Zhou X., Zhang S. (2020). Histamine Induces Microglia Activation and the Release of Proinflammatory Mediators in Rat Brain Via H1R or H4R. J. Neuroimmune Pharmacol..

[185] Zhang S., Zeng X., Yang H., Hu G., He S. (2012). Mast cell tryptase induces microglia activation via protease-activated receptor 2 signaling. Cell. Physiol. Biochem..

[186] Zhou L., Chen L., Li X., Li T., Dong Z., Wang Y.T. (2019). Food allergy induces alteration in brain inflammatory status and cognitive impairments. Behav. Brain Res..

[187] McClain J.L., Mazzotta E.A., Maradiaga N., Duque-Wilckens N., Grants I., Robison A.J., Christofi F.L., Moeser A.J., Gulbransen B.D. (2020). Histamine-dependent interactions between mast cells, glia, and neurons are altered following early-life adversity in mice and humans. Am. J. Physiol. Gastrointest. Liver Physiol..

[188] Galli S.J., Tsai M., Piliponsky A.M. (2008). The development of allergic inflammation. Nature.

[189] Theoharides T.C., Alysandratos K.D., Angelidou A., Delivanis D.A., Sismanopoulos N., Zhang B., Asadi S., Vasiadi M., Weng Z., Miniati A. (2012). Mast cells and inflammation. Biochim. Biophys. Acta.

[190] Mukai K., Tsai M., Saito H., Galli S.J. (2018). Mast cells as sources of cytokines, chemokines, and growth factors. Immunol. Rev..

[191] Gurish M.F., Austen K.F. (2012). Developmental origin and functional specialization of mast cell subsets 1. Immunity.

[192] Olivera A., Beaven M.A., Metcalfe D.D. (2018). Mast cells signal their importance in health and disease. J. Allergy Clin. Immunol..

[193] Theoharides T.C., Valent P., Akin C. (2015). Mast Cells, Mastocytosis, and Related Disorders. N. Engl. J. Med..

[194] Falduto G.H., Pfeiffer A., Luker A., Metcalfe D.D., Olivera A. (2021). Emerging mechanisms contributing to mast cell-mediated pathophysiology with therapeutic implications. Pharmacol. Ther..

[195] Levi-Schaffer F., Gibbs B.F., Hallgren J., Pucillo C., Redegeld F., Siebenhaar F., Vitte J., Mezouar S., Michel M., Puzzovio P.G. (2022). Selected recent advances in understanding the role of human mast cells in health and disease. J. Allergy Clin. Immunol..

[196] Kolkhir P., Elieh-Ali-Komi D., Metz M., Siebenhaar F., Maurer M. (2022). Understanding human mast cells: Lesson from therapies for allergic and non-allergic diseases. Nat. Rev. Immunol..

[197] Dahlin J.S., Maurer M., Metcalfe D.D., Pejler G., Sagi-Eisenberg R., Nilsson G. (2022). The ingenious mast cell: Contemporary insights into mast cell behavior and function. Allergy.

[198] Bawazeer M.A., Theoharides T.C. (2019). IL-33 stimulates human mast cell release of CCL5 and CCL2 via MAPK and NF-kappaB, inhibited by methoxyluteolin. Eur. J. Pharmacol..

[199] Kandere-Grzybowska K., Letourneau R., Kempuraj D., Donelan J., Poplawski S., Boucher W., Athanassiou A., Theoharides T.C. (2003). IL-1 induces vesicular secretion of IL-6 without degranulation from human mast cells. J. Immunol..

[200] Taracanova A., Alevizos M., Karagkouni A., Weng Z., Norwitz E., Conti P., Leeman S.E., Theoharides T.C. (2017). SP and IL-33 together markedly enhance TNF synthesis and secretion from human mast cells mediated by the interaction of their receptors. Proc. Natl. Acad. Sci. USA.

[201] Theoharides T.C., Petra A.I., Taracanova A., Panagiotidou S., Conti P. (2015). Targeting IL-33 in autoimmunity and inflammation. J. Pharmacol. Exp. Ther..

[202] Liew F.Y., Pitman N.I., McInnes I.B. (2010). Disease-associated functions of IL-33: The new kid in the IL-1 family. Nat. Rev. Immunol..

[203] Theoharides T.C., Leeman S.E. (2019). Effect of IL-33 on de novo synthesized mediators from human mast cells. J. Allergy Clin. Immunol..

[204] Saluja R., Khan M., Church M.K., Maurer M. (2015). The role of IL-33 and mast cells in allergy and inflammation. Clin. Transl. Allergy.

[205] Zhang B., Asadi S., Weng Z., Sismanopoulos N., Theoharides T.C. (2012). Stimulated human mast cells secrete mitochondrial components that have autocrine and paracrine inflammatory actions. PLoS ONE.

[206] Collins L.V., Hajizadeh S., Holme E., Jonsson I.M., Tarkowski A. (2004). Endogenously oxidized mitochondrial DNA induces in vivo and in vitro inflammatory responses. J. Leukoc. Biol..

[207] Sun S., Sursal T., Adibnia Y., Zhao C., Zheng Y., Li H., Otterbein L.E., Hauser C.J., Itagaki K. (2013). Mitochondrial DAMPs increase endothelial permeability through neutrophil dependent and independent pathways. PLoS ONE.

[208] Traina G. (2017). Mast cells in the brain—Old cells, new target. J. Integr. Neurosci..

[209] Rozniecki J.J., Dimitriadou V., Lambracht-Hall M., Pang X., Theoharides T.C. (1999). Morphological and functional demonstration of rat dura mater mast cell-neuron interactions in vitro and in vivo. Brain Res..

[210] Theoharides T.C., Konstantinidou A.D. (2007). Corticotropin-releasing hormone and the blood-brain-barrier. Front. Biosci..

[211] Dimitriadou V., Rouleau A., Trung Tuong M.D., Newlands G.J., Miller H.R., Luffau G., Schwartz J.C., Garbarg M. (1997). Functional relationships between sensory nerve fibers and mast cells of dura mater in normal and inflammatory conditions. Neuroscience.

[212] Torrealba F., Riveros M.E., Contreras M., Valdes J.L. (2012). Histamine and motivation. Front. Syst. Neurosci..

[213] Nomura H., Shimizume R., Ikegaya Y. (2021). Histamine: A Key Neuromodulator of Memory Consolidation and Retrieval. Curr. Top. Behav. Neurosci..

[214] Moura D.S., Sultan S., Georgin-Lavialle S., Barete S., Lortholary O., Gaillard R., Hermine O. (2012). Evidence for cognitive impairment in mastocytosis: Prevalence, features and correlations to depression. PLoS ONE.

[215] Spolak-Bobryk N., Romantowski J., Kujawska-Danecka H., Niedoszytko M. (2022). Mastocytosis patients’ cognitive dysfunctions correlate with the presence of spindle-shaped mast cells in bone marrow. Clin. Transl. Allergy.

[216] Boddaert N., Salvador A., Chandesris M.O., Lemaitre H., Grevent D., Gauthier C., Naggara O., Georgin-Lavialle S., Moura D.S., Munsch F. (2017). Neuroimaging evidence of brain abnormalities in mastocytosis. Transl. Psychiatry.

[217] Spath-Schwalbe E., Born J., Schrezenmeier H., Bornstein S.R., Stromeyer P., Drechsler S., Fehm H.L., Porzsolt F. (1994). Interleukin-6 stimulates the hypothalamus-pituitary-adrenocortical axis in man. J. Clin. Endocrinol. Metab..

[218] Theoharides T.C. (2020). Effect of Stress on Neuroimmune Processes. Clin. Ther..

[219] Esposito P., Chandler N., Kandere K., Basu S., Jacobson S., Connolly R., Tutor D., Theoharides T.C. (2002). Corticotropin-releasing hormone and brain mast cells regulate blood-brain-barrier permeability induced by acute stress. J. Pharmacol. Exp. Ther..

[220] Fiorentino M., Sapone A., Senger S., Camhi S.S., Kadzielski S.M., Buie T.M., Kelly D.L., Cascella N., Fasano A. (2016). Blood-brain barrier and intestinal epithelial barrier alterations in autism spectrum disorders. Mol. Autism.

[221] Rozniecki J.J., Sahagian G.G., Kempuraj D., Tao K., Jocobson S., Zhang B., Theoharides T.C. (2010). Brain metastases of mouse mammary adenocarcinoma is increased by acute stress. Brain Res..

[222] Theoharides T.C., Rozniecki J.J., Sahagian G., Jocobson S., Kempuraj D., Conti P., Kalogeromitros D. (2008). Impact of stress and mast cells on brain metastases. J. Neuroimmunol..

[223] Abbott N.J. (2000). Inflammatory mediators and modulation of blood-brain barrier permeability. Cell Mol. Neurobiol..

[224] Pan W., Stone K.P., Hsuchou H., Manda V.K., Zhang Y., Kastin A.J. (2011). Cytokine signaling modulates blood-brain barrier function. Curr. Pharm. Des..

[225] Sayed B.A., Christy A.L., Walker M.E., Brown M.A. (2010). Meningeal mast cells affect early T cell central nervous system infiltration and blood-brain barrier integrity through TNF: A role for neutrophil recruitment?. J. Immunol..

[226] Skaper S.D. (2017). Impact of Inflammation on the Blood-Neural Barrier and Blood-Nerve Interface: From Review to Therapeutic Preview. Int. Rev. Neurobiol..

[227] Sibilano R., Frossi B., Pucillo C.E. (2014). Mast cell activation: A complex interplay of positive and negative signaling pathways. Eur. J. Immunol..

[228] Xu H., Bin N.R., Sugita S. (2018). Diverse exocytic pathways for mast cell mediators. Biochem. Soc. Trans..

[229] Gilfillan A.M., Tkaczyk C. (2006). Integrated signalling pathways for mast-cell activation. Nat. Rev. Immunol.

[230] Gaudenzio N., Sibilano R., Marichal T., Starkl P., Reber L.L., Cenac N., McNeil B.D., Dong X., Hernandez J.D., Sagi-Eisenberg R. (2016). Different activation signals induce distinct mast cell degranulation strategies. J. Clin. Investig..

[231] Theoharides T.C. (2017). Neuroendocrinology of mast cells: Challenges and controversies. Exp. Dermatol..

[232] Theoharides T.C., Tsilioni I., Bawazeer M. (2019). Mast Cells, Neuroinflammation and Pain in Fibromyalgia Syndrome. Front. Cell. Neurosci..

[233] Xu H., Shi X., Li X., Zou J., Zhou C., Liu W., Shao H., Chen H., Shi L. (2020). Neurotransmitter and neuropeptide regulation of mast cell function: A systematic review. J. Neuroinflamm..

[234] Sumpter T.L., Ho C.H., Pleet A.R., Tkacheva O.A., Shufesky W.J., Rojas-Canales D.M., Morelli A.E., Larregina A.T. (2015). Autocrine hemokinin-1 functions as an endogenous adjuvant for IgE-mediated mast cell inflammatory responses. J. Allergy Clin. Immunol..

[235] Levi-Montalcini R., Skaper S.D., Dal Toso R., Petrelli L., Leon A. (1996). Nerve growth factor: From neurotrophin to neurokine. Trends Neurosci..

[236] Donelan J., Boucher W., Papadopoulou N., Lytinas M., Papaliodis D., Dobner P., Theoharides T.C. (2006). Corticotropin-releasing hormone induces skin vascular permeability through a neurotensin-dependent process. Proc. Natl. Acad. Sci. USA.

[237] Theoharides T.C., Zhang B., Kempuraj D., Tagen M., Vasiadi M., Angelidou A., Alysandratos K.D., Kalogeromitros D., Asadi S., Stavrianeas N. (2010). IL-33 augments substance P-induced VEGF secretion from human mast cells and is increased in psoriatic skin. Proc. Natl. Acad. Sci. USA.

[238] Theoharides T.C., Betchaku T., Douglas W.W. (1981). Somatostatin-induced histamine secretion in mast cells. Characterization of the effect. Eur. J. Pharmacol..

[239] Theoharides T.C., Douglas W.W. (1981). Mast cell histamine secretion in response to somatostatin analogues: Structural considerations. Eur. J. Pharmacol..

[240] Theoharides T.C., Papaliodis D., Tagen M., Konstantinidou A., Kempuraj D., Clemons A. (2005). Chronic fatigue syndrome, mast cells, and tricyclic antidepressants. J. Clin. Psychopharmacol..

[241] Gordon J.R., Galli S.J. (1990). Mast cells as a source of both preformed and immunologically inducible TNF-alpha/cachectin. Nature.

[242] Zhang B., Alysandratos K.D., Angelidou A., Asadi S., Sismanopoulos N., Delivanis D.A., Weng Z., Miniati A., Vasiadi M., Katsarou-Katsari A. (2011). Human mast cell degranulation and preformed TNF secretion require mitochondrial translocation to exocytosis sites: Relevance to atopic dermatitis. J. Allergy Clin. Immunol..

[243] Taracanova A., Tsilioni I., Conti P., Norwitz E.R., Leeman S.E., Theoharides T.C. (2018). Substance P and IL-33 administered together stimulate a marked secretion of IL-1beta from human mast cells, inhibited by methoxyluteolin. Proc. Natl. Acad. Sci. USA.

[244] Yu Y., Blokhuis B.R., Garssen J., Redegeld F.A. (2016). Non-IgE mediated mast cell activation. Eur. J. Pharmacol..

[245] Theoharides T.C., Kempuraj D., Tagen M., Conti P., Kalogeromitros D. (2007). Differential release of mast cell mediators and the pathogenesis of inflammation. Immunol. Rev..

[246] Gagari E., Tsai M., Lantz C.S., Fox L.G., Galli S.J. (1997). Differential release of mast cell interleukin-6 via c-kit. Blood.

[247] Theoharides T.C., Boucher W., Spear K. (2002). Serum interleukin-6 reflects disease severity and osteoporosis in mastocytosis patients. Int. Arch. Allergy Immunol..

[248] Brockow K., Akin C., Huber M., Metcalfe D.D. (2005). IL-6 levels predict disease variant and extent of organ involvement in patients with mastocytosis. Clin. Immunol..

[249] Mayado A., Teodosio C., Garcia-Montero A.C., Matito A., Rodriguez-Caballero A., Morgado J.M., Muniz C., Jara-Acevedo M., Alvarez-Twose I., Sanchez-Munoz L. (2016). Increased IL6 plasma levels in indolent systemic mastocytosis patients are associated with high risk of disease progression. Leukemia.

[250] Kaur D., Gomez E., Doe C., Berair R., Woodman L., Saunders R., Hollins F., Rose F.R., Amrani Y., May R. (2015). IL-33 drives airway hyper-responsiveness through IL-13-mediated mast cell: Airway smooth muscle crosstalk. Allergy.

[251] Abraham S.N., St John A.L. (2010). Mast cell-orchestrated immunity to pathogens. Nat. Rev. Immunol..

[252] Song S.T., Wu M.L., Zhang H.J., Su X., Wang J.H. (2022). Mast Cell Activation Triggered by Retrovirus Promotes Acute Viral Infection. Front. Microbiol..

[253] Gebremeskel S., Schanin J., Coyle K.M., Butuci M., Luu T., Brock E.C., Xu A., Wong A., Leung J., Korver W. (2021). Mast Cell and Eosinophil Activation Are Associated With COVID-19 and TLR-Mediated Viral Inflammation: Implications for an Anti-Siglec-8 Antibody. Front. Immunol..

[254] Motta Junior J.D.S., Miggiolaro A., Nagashima S., de Paula C.B.V., Baena C.P., Scharfstein J., de Noronha L. (2020). Mast Cells in Alveolar Septa of COVID-19 Patients: A Pathogenic Pathway That May Link Interstitial Edema to Immunothrombosis. Front. Immunol..

[255] Wu M.L., Liu F.L., Sun J., Li X., He X.Y., Zheng H.Y., Zhou Y.H., Yan Q., Chen L., Yu G.Y. (2021). SARS-CoV-2-triggered mast cell rapid degranulation induces alveolar epithelial inflammation and lung injury. Signal Transduct. Target. Ther..

[256] Tan J., Anderson D.E., Rathore A.P.S., O’Neill A., Mantri C.K., Saron W.A.A., Lee C., Cui C.W., Kang A.E.Z., Foo R. (2021). Signatures of mast cell activation are associated with severe COVID-19. medRxiv.

[257] Zelechowska P., Brzezinska-Blaszczyk E., Agier J., Kozlowska E. (2022). Different effectiveness of fungal pathogen-associated molecular patterns (PAMPs) in activating rat peritoneal mast cells. Immunol. Lett..

[258] Krysko O., Bourne J.H., Kondakova E., Galova E.A., Whitworth K., Newby M.L., Bachert C., Hill H., Crispin M., Stamataki Z. (2022). Severity of SARS-CoV-2 infection is associated with high numbers of alveolar mast cells and their degranulation. Front. Immunol..

[259] Takagi D., Ishiyama K., Suganami M., Ushijima T., Fujii T., Tazoe Y., Kawasaki M., Noguchi K., Makino A. (2021). Manganese toxicity disrupts indole acetic acid homeostasis and suppresses the CO(2) assimilation reaction in rice leaves. Sci. Rep..

[260] Wechsler J.B., Butuci M., Wong A., Kamboj A.P., Youngblood B.A. (2022). Mast cell activation is associated with post-acute COVID-19 syndrome. Allergy.

[261] da Silveira Gorman R., Syed I.U. (2022). Connecting the Dots in Emerging Mast Cell Research: Do Factors Affecting Mast Cell Activation Provide a Missing Link between Adverse COVID-19 Outcomes and the Social Determinants of Health?. Med. Sci..

[262] Scozzi D., Cano M., Ma L., Zhou D., Zhu J.H., O’Halloran J.A., Goss C., Rauseo A.M., Liu Z., Sahu S.K. (2021). Circulating mitochondrial DNA is an early indicator of severe illness and mortality from COVID-19. JCI Insight.

[263] Keykavousi K., Nourbakhsh F., Abdollahpour N., Fazeli F., Sedaghat A., Soheili V., Sahebkar A. (2022). A Review of Routine Laboratory Biomarkers for the Detection of Severe COVID-19 Disease. Int. J. Anal. Chem..

[264] DeKosky S.T., Kochanek P.M., Valadka A.B., Clark R.S.B., Chou S.H., Au A.K., Horvat C., Jha R.M., Mannix R., Wisniewski S.R. (2021). Blood Biomarkers for Detection of Brain Injury in COVID-19 Patients. J. Neurotrauma.

[265] Frontera J.A., Boutajangout A., Masurkar A.V., Betensky R.A., Ge Y., Vedvyas A., Debure L., Moreira A., Lewis A., Huang J. (2022). Comparison of serum neurodegenerative biomarkers among hospitalized COVID-19 patients versus non-COVID subjects with normal cognition, mild cognitive impairment, or Alzheimer’s dementia. Alzheimers Dement..

[266] Wang Z., Waldman M.F., Basavanhally T.J., Jacobs A.R., Lopez G., Perichon R.Y., Ma J.J., Mackenzie E.M., Healy J.B., Wang Y. (2022). Autoimmune gene expression profiling of fingerstick whole blood in Chronic Fatigue Syndrome. J. Transl. Med..

[267] Kandikattu H.K., Venkateshaiah S.U., Kumar S., Mishra A. (2020). IL-15 immunotherapy is a viable strategy for COVID-19. Cytokine Growth Factor Rev..

[268] Lu T., Ma R., Dong W., Teng K.Y., Kollath D.S., Li Z., Yi J., Bustillos C., Ma S., Tian L. (2022). Off-the-shelf CAR natural killer cells secreting IL-15 target spike in treating COVID-19. Nat. Commun..

[269] Kassianidis G., Siampanos A., Poulakou G., Adamis G., Rapti A., Milionis H., Dalekos G.N., Petrakis V., Sympardi S., Metallidis S. (2022). Calprotectin and Imbalances between Acute-Phase Mediators Are Associated with Critical Illness in COVID-19. Int. J. Mol. Sci..

[270] Yasuda K., Nakanishi K., Tsutsui H. (2019). Interleukin-18 in Health and Disease. Int. J. Mol. Sci..

[271] Ihim S.A., Abubakar S.D., Zian Z., Sasaki T., Saffarioun M., Maleknia S., Azizi G. (2022). Interleukin-18 cytokine in immunity, inflammation, and autoimmunity: Biological role in induction, regulation, and treatment. Front. Immunol..

[272] Wu M., Xu L., Wang Y., Zhou N., Zhen F., Zhang Y., Qu X., Fan H., Liu S., Chen Y. (2018). S100A8/A9 induces microglia activation and promotes the apoptosis of oligodendrocyte precursor cells by activating the NF-kappaB signaling pathway. Brain Res. Bull..

[273] Berg-Hansen P., Vandvik B., Fagerhol M., Holmoy T. (2009). Calprotectin levels in the cerebrospinal fluid reflect disease activity in multiple sclerosis. J. Neuroimmunol..

[274] Stascheit F., Hotter B., Klose S., Meisel C., Meisel A., Klehmet J. (2021). Calprotectin in Chronic Inflammatory Demyelinating Polyneuropathy and Variants-A Potential Novel Biomarker of Disease Activity. Front. Neurol..

[275] Sudhof T.C. (2008). Neuroligins and neurexins link synaptic function to cognitive disease. Nature.

[276] Camporesi E., Lashley T., Gobom J., Lantero-Rodriguez J., Hansson O., Zetterberg H., Blennow K., Becker B. (2021). Neuroligin-1 in brain and CSF of neurodegenerative disorders: Investigation for synaptic biomarkers. Acta Neuropathol. Commun..

[277] Dufort-Gervais J., Provost C., Charbonneau L., Norris C.M., Calon F., Mongrain V., Brouillette J. (2020). Neuroligin-1 is altered in the hippocampus of Alzheimer’s disease patients and mouse models, and modulates the toxicity of amyloid-beta oligomers. Sci. Rep..

[278] Zhang K., Gao X., Qi H., Li J., Zheng Z., Zhang F. (2010). Gender differences in cognitive ability associated with genetic variants of NLGN4. Neuropsychobiology.

[279] Cantuti-Castelvetri L., Ojha R., Pedro L.D., Djannatian M., Franz J., Kuivanen S., van der Meer F., Kallio K., Kaya T., Anastasina M. (2020). Neuropilin-1 facilitates SARS-CoV-2 cell entry and infectivity. Science.

[280] Aceti A., Margarucci L.M., Scaramucci E., Orsini M., Salerno G., Di Sante G., Gianfranceschi G., Di Liddo R., Valeriani F., Ria F. (2020). Serum S100B protein as a marker of severity in COVID-19 patients. Sci. Rep..

[281] Zhou S., Zhu W., Zhang Y., Pan S., Bao J. (2018). S100B promotes microglia M1 polarization and migration to aggravate cerebral ischemia. Inflamm. Res..

[282] Xu J., Wang H., Won S.J., Basu J., Kapfhamer D., Swanson R.A. (2016). Microglial activation induced by the alarmin S100B is regulated by poly(ADP-ribose) polymerase-1. Glia.

[283] Bianchi R., Kastrisianaki E., Giambanco I., Donato R. (2011). S100B protein stimulates microglia migration via RAGE-dependent up-regulation of chemokine expression and release. J. Biol. Chem..

[284] Hopman J.H., Santing J.A.L., Foks K.A., Verheul R.J., van der Linden C.M., van den Brand C.L., Jellema K. (2023). Biomarker S100B in plasma a screening tool for mild traumatic brain injury in an emergency department. Brain Inj.

[285] Shahim P., Politis A., van der Merwe A., Moore B., Chou Y.Y., Pham D.L., Butman J.A., Diaz-Arrastia R., Gill J.M., Brody D.L. (2020). Neurofilament light as a biomarker in traumatic brain injury. Neurology.

[286] Savarraj J., Park E.S., Colpo G.D., Hinds S.N., Morales D., Ahnstedt H., Paz A.S., Assing A., Liu F., Juneja S. (2021). Brain injury, endothelial injury and inflammatory markers are elevated and express sex-specific alterations after COVID-19. J. Neuroinflamm..

[287] Park D., Joo S.S., Lee H.J., Choi K.C., Kim S.U., Kim Y.B. (2012). Microtubule-associated protein 2, an early blood marker of ischemic brain injury. J. Neurosci. Res..

[288] Hicks C., Dhiman A., Barrymore C., Goswami T. (2022). Traumatic Brain Injury Biomarkers, Simulations and Kinetics. Bioengineering.

[289] Iaffaldano P., Ruggieri M., Viterbo R.G., Mastrapasqua M., Trojano M. (2014). The improvement of cognitive functions is associated with a decrease of plasma Osteopontin levels in Natalizumab treated relapsing multiple sclerosis. Brain Behav. Immun..

[290] Chai Y.L., Chong J.R., Raquib A.R., Xu X., Hilal S., Venketasubramanian N., Tan B.Y., Kumar A.P., Sethi G., Chen C.P. (2021). Plasma osteopontin as a biomarker of Alzheimer’s disease and vascular cognitive impairment. Sci. Rep..

[291] Khalifa S., Holmstead R.L., Casida J.E. (1976). Toxaphene degradation by iron(II) protoporphyrin systems. J. Agric. Food Chem..

[292] Fernandez-Castaneda A., Lu P., Geraghty A.C., Song E., Lee M.H., Wood J., O’Dea M.R., Dutton S., Shamardani K., Nwangwu K. (2022). Mild respiratory COVID can cause multi-lineage neural cell and myelin dysregulation. Cell.

[293] Nazarinia D., Behzadifard M., Gholampour J., Karimi R., Gholampour M. (2022). Eotaxin-1 (CCL11) in neuroinflammatory disorders and possible role in COVID-19 neurologic complications. Acta Neurol. Belg..

[294] Spitzer D., Guerit S., Puetz T., Khel M.I., Armbrust M., Dunst M., Macas J., Zinke J., Devraj G., Jia X. (2022). Profiling the neurovascular unit unveils detrimental effects of osteopontin on the blood-brain barrier in acute ischemic stroke. Acta Neuropathol..

[295] Yan Y., Chen R., Wang X., Hu K., Huang L., Lu M., Hu Q. (2019). CCL19 and CCR7 Expression, Signaling Pathways, and Adjuvant Functions in Viral Infection and Prevention. Front. Cell. Dev. Biol..

[296] Tveita A., Murphy S.L., Holter J.C., Kildal A.B., Michelsen A.E., Lerum T.V., Kaarbo M., Heggelund L., Holten A.R., Finbraten A.K. (2022). High circulating levels of the homeostatic chemokines CCL19 and CCL21 predict mortality and disease severity in COVID-19. J. Infect. Dis..

[297] Kushner P., McCarberg B.H., Grange L., Kolosov A., Haveric A.L., Zucal V., Petruschke R., Bissonnette S. (2022). The use of non-steroidal anti-inflammatory drugs (NSAIDs) in COVID-19. NPJ Prim. Care Respir. Med..

[298] Bicker J., Alves G., Fonseca C., Falcao A., Fortuna A. (2020). Repairing blood-CNS barriers: Future therapeutic approaches for neuropsychiatric disorders. Pharmacol. Res..

[299] Alegre-Del-Rey E.J., Fenix-Caballero S., Salmeron-Navas F.J., Gil-Sierra M.D., Sierra-Sanchez J.F., Diaz-Alersi Rosety R.L. (2022). Systematic review and meta-analysis of interleulin-6 inhibitors in reducing mortality for hospitalized patients with COVID-19. Farm. Hosp..

[300] Dimopoulos G., de Mast Q., Markou N., Theodorakopoulou M., Komnos A., Mouktaroudi M., Netea M.G., Spyridopoulos T., Verheggen R.J., Hoogerwerf J. (2020). Favorable Anakinra Responses in Severe COVID-19 Patients with Secondary Hemophagocytic Lymphohistiocytosis. Cell Host Microbe.

[301] Liu J., Dong J., Yu Y., Yang X., Shu J., Bao H. (2022). Corticosteroids showed more efficacy in treating hospitalized patients with COVID-19 than standard care but the effect is minimal: A systematic review and meta-analysis. Front. Public Health.

[302] Cheng B., Ma J., Yang Y., Shao T., Zhao B., Zeng L. (2021). Systemic Corticosteroid Administration in Coronavirus Disease 2019 Outcomes: An Umbrella Meta-Analysis Incorporating Both Mild and Pulmonary Fibrosis-Manifested Severe Disease. Front. Pharmacol..

[303] Wagner C., Griesel M., Mikolajewska A., Metzendorf M.I., Fischer A.L., Stegemann M., Spagl M., Nair A.A., Daniel J., Fichtner F. (2022). Systemic corticosteroids for the treatment of COVID-19: Equity-related analyses and update on evidence. Cochrane Database Syst. Rev..

[304] Torres A., Motos A., Cilloniz C., Ceccato A., Fernandez-Barat L., Gabarrus A., Bermejo-Martin J., Ferrer R., Riera J., Perez-Arnal R. (2022). Major candidate variables to guide personalised treatment with steroids in critically ill patients with COVID-19: CIBERESUCICOVID study. Intensive Care Med..

[305] Mustafa S.S. (2023). Steroid induced secondary immune deficiency. Ann. Allergy Asthma Immunol..

[306] Theoharides T.C., Conti P. (2020). Dexamethasone for COVID-19? Not so fast. J. Biol. Regul. Homeost. Agents.

[307] Middleton E., Kandaswami C., Theoharides T.C. (2000). The effects of plant flavonoids on mammalian cells: Implications for inflammation, heart disease, and cancer. Pharmacol. Rev..

[308] Leyva-Lopez N., Gutierrez-Grijalva E.P., mbriz-Perez D.L., Heredia J.B. (2016). Flavonoids as Cytokine Modulators: A Possible Therapy for Inflammation-Related Diseases 1. Int. J. Mol. Sci..

[309] Jager A.K., Saaby L. (2011). Flavonoids and the CNS. Molecules.

[310] Calfio C., Gonzalez A., Singh S.K., Rojo L.E., Maccioni R.B. (2020). The Emerging Role of Nutraceuticals and Phytochemicals in the Prevention and Treatment of Alzheimer’s Disease. J. Alzheimers Dis..

[311] Kempuraj D., Thangavel R., Kempuraj D.D., Ahmed M.E., Selvakumar G.P., Raikwar S.P., Zaheer S.A., Iyer S.S., Govindarajan R., Chandrasekaran P.N. (2021). Neuroprotective effects of flavone luteolin in neuroinflammation and neurotrauma. Biofactors.

[312] Calis Z., Mogulkoc R., Baltaci A.K. (2020). The Roles of Flavonols/Flavonoids in Neurodegeneration and Neuroinflammation. Mini Rev. Med. Chem..

[313] Rezai-Zadeh K., Ehrhart J., Bai Y., Sanberg P.R., Bickford P., Tan J., Shytle R.D. (2008). Apigenin and luteolin modulate microglial activation via inhibition of STAT1-induced CD40 expression. J. Neuroinflamm..

[314] Jang S., Kelley K.W., Johnson R.W. (2008). Luteolin reduces IL-6 production in microglia by inhibiting JNK phosphorylation and activation of AP-1. Proc. Natl. Acad. Sci. USA.

[315] Weng Z., Patel A.B., Panagiotidou S., Theoharides T.C. (2015). The novel flavone tetramethoxyluteolin is a potent inhibitor of human mast cells. J. Allergy Clin. Immunol..

[316] Seelinger G., Merfort I., Schempp C.M. (2008). Anti-oxidant, anti-inflammatory and anti-allergic activities of luteolin. Planta Med..

[317] Theoharides T.C., Conti P., Economu M. (2014). Brain inflammation, neuropsychiatric disorders, and immunoendocrine effects of luteolin. J. Clin. Psychopharmacol..

[318] Ashaari Z., Hadjzadeh M.A., Hassanzadeh G., Alizamir T., Yousefi B., Keshavarzi Z., Mokhtari T. (2018). The Flavone Luteolin Improves Central Nervous System Disorders by Different Mechanisms: A Review. J. Mol. Neurosci..

[319] Dajas F., Rivera-Megret F., Blasina F., Arredondo F., bin-Carriquiry J.A., Costa G., Echeverry C., Lafon L., Heizen H., Ferreira M. (2003). Neuroprotection by flavonoids 1. Braz. J. Med. Biol. Res..

[320] Lin T.Y., Lu C.W., Wang S.J. (2016). Luteolin protects the hippocampus against neuron impairments induced by kainic acid in rats. NeuroToxicology.

[321] Zhu L.H., Bi W., Qi R.B., Wang H.D., Lu D.X. (2011). Luteolin inhibits microglial inflammation and improves neuron survival against inflammation. Int. J. Neurosci..

[322] Rezai-Zadeh K., Douglas S.R., Bai Y., Tian J., Hou H., Mori T., Zeng J., Obregon D., Town T., Tan J. (2009). Flavonoid-mediated presenilin-1 phosphorylation reduces Alzheimer’s disease beta-amyloid production. J. Cell. Mol. Med..

[323] Yao Z.H., Yao X.L., Zhang Y., Zhang S.F., Hu J.C. (2018). Luteolin Could Improve Cognitive Dysfunction by Inhibiting Neuroinflammation. Neurochem. Res..

[324] Gratton G., Weaver S.R., Burley C.V., Low K.A., Maclin E.L., Johns P.W., Pham Q.S., Lucas S.J.E., Fabiani M., Rendeiro C. (2020). Dietary flavanols improve cerebral cortical oxygenation and cognition in healthy adults. Sci. Rep..

[325] Devi S.A., Chamoli A. (2020). Polyphenols as an Effective Therapeutic Intervention Against Cognitive Decline During Normal and Pathological Brain Aging. Adv. Exp. Med. Biol..

[326] Theoharides T.C., Stewart J.M., Hatziagelaki E., Kolaitis G. (2015). Brain “fog,” inflammation and obesity: Key aspects of 2 neuropsychiatric disorders improved by luteolin. Front. Neurosci..

[327] Stefano G.B., Buttiker P., Weissenberger S., Martin A., Ptacek R., Kream R.M. (2021). Editorial: The Pathogenesis of Long-Term Neuropsychiatric COVID-19 and the Role of Microglia, Mitochondria, and Persistent Neuroinflammation: A Hypothesis. Med. Sci. Monit..

[328] Hugon J., Msika E.F., Queneau M., Farid K., Paquet C. (2022). Long COVID: Cognitive complaints (brain fog) and dysfunction of the cingulate cortex. J. Neurol..

[329] Theoharides T.C., Guerra L., Patel K. (2022). Successful Treatment of a Patient With Severe COVID-19 Using an Integrated Approach Addressing Mast Cells and Their Mediators. Int. J. Infect. Dis..

[330] Islam A., Islam M.S., Rahman M.K., Uddin M.N., Akanda M.R. (2020). The pharmacological and biological roles of eriodictyol. Arch. Pharm. Res..

[331] Zhang L., Liu C., Yuan M. (2020). Eriodictyol produces antidepressant-like effects and ameliorates cognitive impairments induced by chronic stress. Neuroreport.

[332] Deng Z., Hassan S., Rafiq M., Li H., He Y., Cai Y., Kang X., Liu Z., Yan T. (2020). Pharmacological Activity of Eriodictyol: The Major Natural Polyphenolic Flavanone. Evid. Based. Complement. Alternat. Med..

[333] Mokdad-Bzeouich I., Mustapha N., Sassi A., Bedoui A., Ghoul M., Ghedira K., Chekir-Ghedira L. (2016). Investigation of immunomodulatory and anti-inflammatory effects of eriodictyol through its cellular anti-oxidant activity. Cell Stress Chaperones.

[334] Deshpande R.R., Tiwari A.P., Nyayanit N., Modak M. (2020). In silico molecular docking analysis for repurposing therapeutics against multiple proteins from SARS-CoV-2. Eur. J. Pharmacol..

[335] Rudrapal M., Issahaku A.R., Agoni C., Bendale A.R., Nagar A., Soliman M.E.S., Lokwani D. (2022). In silico screening of phytopolyphenolics for the identification of bioactive compounds as novel protease inhibitors effective against SARS-CoV-2. J. Biomol. Struct. Dyn..

[336] Ton A.T., Gentile F., Hsing M., Ban F., Cherkasov A. (2020). Rapid Identification of Potential Inhibitors of SARS-CoV-2 Main Protease by Deep Docking of 1.3 Billion Compounds. Mol. Inform..

[337] Gentile F., Fernandez M., Ban F., Ton A.T., Mslati H., Perez C.F., Leblanc E., Yaacoub J.C., Gleave J., Stern A. (2021). Automated discovery of noncovalent inhibitors of SARS-CoV-2 main protease by consensus Deep Docking of 40 billion small molecules. Chem. Sci..

[338] Vijayan R., Gourinath S. (2021). Structure-based inhibitor screening of natural products against NSP15 of SARS-CoV-2 revealed thymopentin and oleuropein as potent inhibitors. J. Proteins Proteom..

[339] Abdelgawad S.M., Hassab M.A.E., Abourehab M.A.S., Elkaeed E.B., Eldehna W.M. (2022). Olive Leaves as a Potential Phytotherapy in the Treatment of COVID-19 Disease; A Mini-Review. Front. Pharmacol..

[340] Geromichalou E.G., Geromichalos G.D. (2022). In Silico Approach for the Evaluation of the Potential Antiviral Activity of Extra Virgin Olive Oil (EVOO) Bioactive Constituents Oleuropein and Oleocanthal on Spike Therapeutic Drug Target of SARS-CoV-2. Molecules.

[341] Ordonez A.A., Bullen C.K., Villabona-Rueda A.F., Thompson E.A., Turner M.L., Merino V.F., Yan Y., Kim J., Davis S.L., Komm O. (2022). Sulforaphane exhibits antiviral activity against pandemic SARS-CoV-2 and seasonal HCoV-OC43 coronaviruses in vitro and in mice. Commun. Biol..

